# *Borrelia miyamotoi* and *Borrelia burgdorferi* (*sensu lato*) identification and survey of tick-borne encephalitis virus in ticks from north-eastern Germany

**DOI:** 10.1186/s13071-020-3969-7

**Published:** 2020-02-27

**Authors:** Cristian Răileanu, Oliver Tauchmann, Ana Vasić, Elisabeth Wöhnke, Cornelia Silaghi

**Affiliations:** 1 Institute of Infectology, Friedrich-Loeffler-Institut, Südufer 10, 17943 Greifswald-Insel Riems, Germany; 2 Institute of Molecular Virology and Cell Biology, Friedrich-Loeffler-Institut, Greifswald-Insel Riems, Germany; 3 Department of Biology, University of Greifswald, Domstrasse 11, 17489 Greifswald, Germany

**Keywords:** *Ixodes ricinus*, Lyme borreliosis, *Borrelia miyamotoi*, Tick-borne encephalitis virus, Mecklenburg-Western Pomerania

## Abstract

**Background:**

*Ixodes ricinus* is the most common tick species in Europe and the main vector for *Borrelia burgdorferi* (*sensu lato*) and tick-borne encephalitis virus (TBEV). It is involved also in the transmission of *Borrelia miyamotoi*, a relapsing fever spirochete that causes health disorders in humans. Little is known regarding the circulation of *Borrelia* species and the natural foci of TBEV in north-eastern Germany. The goal of this study was to investigate the infection rates of *Borrelia* spp. and of TBEV in *I. ricinus* ticks from north-eastern Germany.

**Methods:**

Ticks were collected by flagging from 14 forest sites in Mecklenburg-Western Pomerania between April and October 2018. RNA and DNA extraction was performed from individual adult ticks and from pools of 2–10 nymphs. Real time reverse transcription PCR (RT-qPCR) targeted the 3′ non-coding region of TBEV, while DNA of *Borrelia* spp. was tested by nested PCR for the amplification of 16S-23S intergenic spacer. Multilocus sequence typing (MLST) was performed on *B. miyamotoi* isolates.

**Results:**

In total, 2407 ticks were collected (239 females, 232 males and 1936 nymphs). Female and male *I. ricinus* ticks had identical infection rates (both 12.1%) for *Borrelia* spp., while nymphal pools showed a minimum infection rate (MIR) of 3.3%. Sequencing revealed four *Borrelia* species: *B. afzelii*, *B. garinii*, *B. valaisiana* and *B. miyamotoi*. *Borrelia afzelii* had the highest prevalence in adult ticks (5.5%) and nymphs (MIR of 1.8%). *Borrelia miyamotoi* was identified in 3.0% of adults and registered the MIR of 0.8% in nymphs. *Borrelia valaisiana* was confirmed in 2.5% adult ticks and nymphs had the MIR of 0.7%, while *B. garinii* was present in 1.1% of adults and showed a MIR of 0.1% in nymphs. The MLST of *B. miyamotoi* isolates showed that they belong to sequence type 635. No tick sample was positive after RT-qPCR for TBEV RNA.

**Conclusions:**

The prevalence of *B. miyamotoi* in *I. ricinus* ticks registered similar levels to other reports from Europe suggesting that this agent might be well established in the local tick population. The detection of *B. burgdorferi* (*s.l.*) indicates a constant circulation in tick populations from this region.
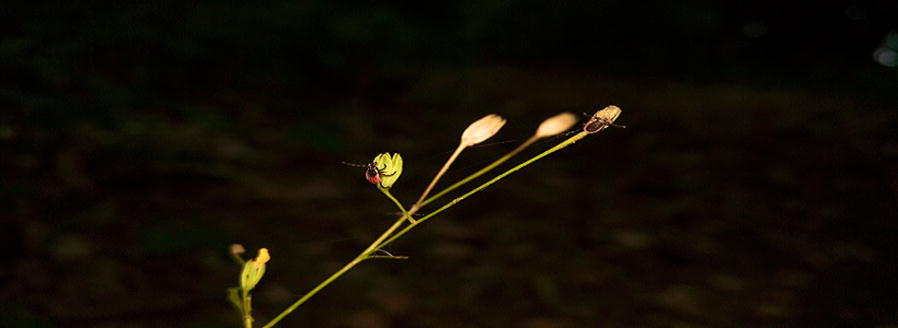

## Background

Ixodid ticks transmit the greatest variety of human and animal pathogens compared to any other arthropod [1]. *Ixodes ricinus* is the most widespread hard tick species in Europe and Germany [2, 3] known to feed on many different vertebrate hosts. The most relevant pathogens for which *I. ricinus* is a competent vector are *Borrelia burgdorferi* (*sensu lato*), the agent responsible for Lyme disease and the tick-borne encephalitis virus (TBEV) [4, 5]. Spirochetes of the *B. burgdorferi* (*s.l.*) complex can cause Lyme borreliosis, the most frequent tick-borne disease of humans in the Northern Hemisphere [6].

In Europe, each year tens of thousands of human cases are being reported [7]. In Germany, Lyme borreliosis is the most common tick-borne disease in humans, registering between 2013 and 2017 mean annual incidence rates on district level from 0.5/100,000 up to 138/100,000 [8]. Currently, 22 genospecies belonging to the *B. burgdorferi* (*s.l.*) complex exist, out of which 11 are circulating in Europe [9]. In humans, Lyme borreliosis is a multi-systemic disease that evolves under several clinical manifestations: erythema migrans (most frequent); Lyme arthritis; Lyme carditis; and neuroborreliosis [10]. Detection of *B. burgdorferi* (*s.l.*) in ticks from Germany was reported in several studies, prevalence rates ranging from 2% up to 36.2% in the southern part of the country [11–19].

*Borrelia miyamotoi* is the only member of the relapsing fever group for which *I. ricinus* represents the main vector in Europe [20], causing influenza-like symptoms in humans characterized by fever, nausea, myalgia, fatigue, headache or chills [21].

Interestingly, information regarding the presence of *B. miyamotoi* in ticks from Northern Germany was lacking until 2018 when the first prevalence for this pathogen was published, 2.1% of *I. ricinus* ticks (8.9% of *Borrelia* spp. positive specimens) collected from the city of Hannover being positive by reverse line blot [16]. Positive *I. ricinus* ticks for *B. miyamotoi* were also detected in Western and Southern Germany, with infection rates varying from 1.8% to 2.7% [11, 17].

Tick-borne encephalitis virus (TBEV) is the main viral agent transmitted by *I. ricinus* ticks in Europe [22]. It belongs to genus *Flavivirus*, family *Flaviviridae* and has three main subtypes: TBE-Eu (European sub-type) circulating in Europe; TBE-FE (Far East) found in Asia; and TBE-Sib (Siberia) located in Russia and reaching also eastern part of Europe [22]. The virus has an endemic occurrence in 27 countries in Europe [23], with the Czech Republic, the Baltic countries, and Slovenia registering the highest annual incidence rates (5–18.6/100,000) [24]. It can cause severe central nervous system infections in humans that can be fatal [25]. Infections with TBEV are mostly located in Southern Germany, 89% of human cases occurring in Bavaria and Baden-Wurttemberg [26].

Mecklenburg-Western Pomerania is considered a low risk area for TBE but isolated cases of infection were reported between 2002 and 2018 [26]. In 2004, the first autochthonous human case was registered after 19 years [27]. Furthermore, detection of TBEV in questing *I. ricinus* was found in 2007 for the first time since 1992 [28]. Later studies found anti-TBEV-IgG antibodies in wild game [29], sheep and goats [30].

The goal of the present study was to investigate the occurrence of *Borrelia* spp. and tick-borne encephalitis virus in *I. ricinus* ticks in Mecklenburg-Western Pomerania. Furthermore, multilocus sequence typing (MLST) for *B. miyamotoi* was performed, a pathogen for which there is still a lack of data regarding its circulation in ticks from Northern Germany.

## Methods

### Study sites and ticks

**Fig. 1:**
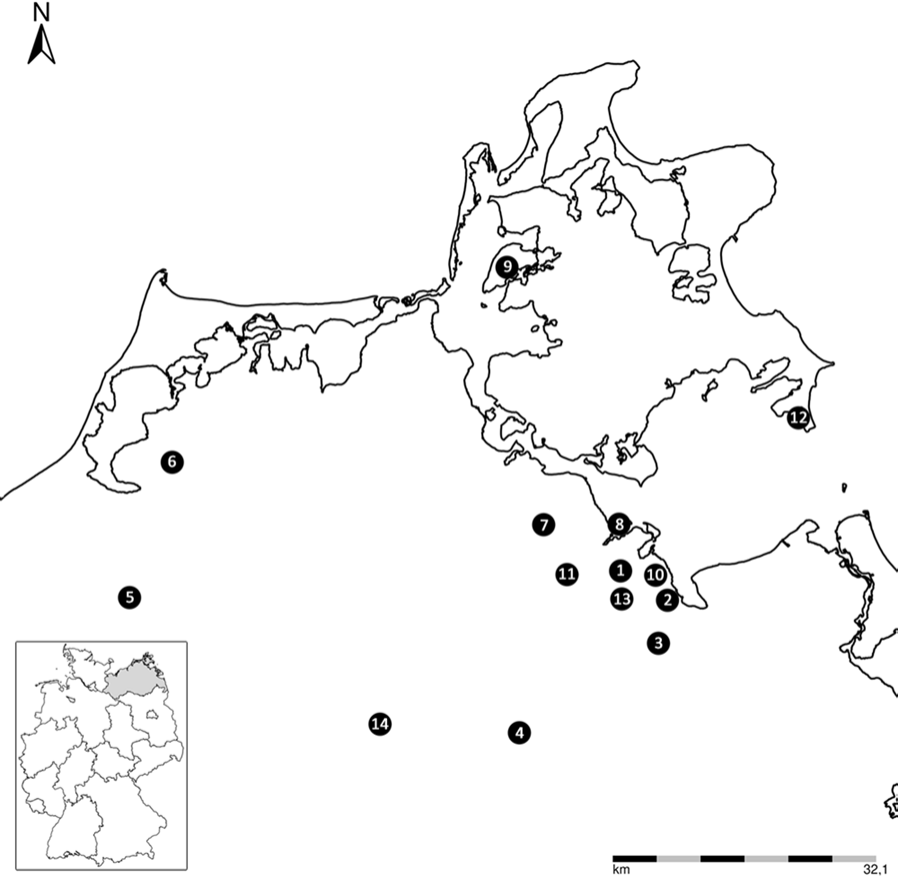
The map of the sampling sites in Mecklenburg-Western Pomerania, Germany. Map: Map Explorer version 2.0 ^®^2010, FLI Wusterhausen

Study sites were 14 forested habitats in the north-eastern part of Mecklenburg-Western Pomerania (12 sites from mainland of Mecklenburg-Western Pomerania and 2 sites from the island of Ruegen; Fig. 1). The tick collection took place from April to October 2018, by flagging a 1 m^2^ white cotton material on the low vegetation and regularly checking it for ticks. The collection was performed monthly in three locations (1, Kieshof; 3, Weitenhagen; and 7, Mannhagen), while for the rest of the sites, ticks were obtained after only one visit (Table 1).

**Table 1:** Number of collected ticks from each study site in Mecklenburg-Western Pomerania

Site	Location	Collected *Ixodes ricinus* ticks				Coordinates
		F	M	N	Total	
1	Kieshof^a^	123	149	1134	1406	54.130256N, 13.345506E
2	Ryck	3	2	10	15	54.095043N, 13.429127E
3	Weitenhagen^a^	79	43	501	623	54.048814N, 13.407368E
4	Loitz	–	1	4	5	53.96054N, 13.13994E
5	Carlsruhe	–	–	7	7	54.13068N, 12.43077E
6	Tempel	–	3	33	36	53.96054N, 13.13994E
7	Mannhagen^a^	14	7	128	149	54.185725N, 13.207810E
8	Riems	9	17	4	30	54.181194N, 13.347872E
9	Ummanz	1	–	36	37	54.468392N, 13.167908E
10	Ladebow	–	1	12	13	54.113863N, 13.416879E
11	Horst	8	6	8	22	54.129832N, 13.245665E
12	Thiesow	1	3	54	58	54.284438N, 13.694322E
13	Wackerow	1	–	–	1	53.96054N, 13.13994E
14	Annenhof	–	–	5	5	53.96054N, 13.13994E
Total		239	232	1936	2407	

^a^Monthly collection of ticks from April to October 2018

*Abbreviations*: F, females; M, males; N, nymphs; –, no collected ticks

### Processing tick samples

After collection, ticks were transferred alive to the laboratory. We performed two washing steps of ticks; first washing in 70% ethanol for 5 min, followed by a second wash in a distilled water bath for 5 min. Ticks were dried with paper tissues and then were subjected to morphological identification using taxonomical identification keys [31]. After identification, ticks were stored at − 80 °C until further analysis. Homogenization of ticks was performed from each individual adult tick and from pools of nymphs (2 to 10 nymphs in each pool). Nymphs were pooled based on the collection site. Tick lysis was performed in a 2 ml tube, using one 5 mm metal bead and 300 µl modified minimum essential medium (Collection of Cell Lines in Veterinary Medicine, Friedrich-Loeffler-Institut, Germany) supplemented with 10% fetal calf serum. Homogenization was achieved using a TissueLyser II (Qiagen, Venlo, Netherlands) at 30 Hz, repeated twice for 1 min. Following tick lysis, each tube was centrifuged at 1500× *rpm* for 2 min. DNA and RNA extraction was performed from 100 µl aliquots with GeneJET Genomic DNA Purification kit and GeneJET RNA Purification Kit, respectively, according to the manufacturer instructions (Thermo Fisher Scientific, Waltham, USA). Purified RNA and DNA were eluted in 100 µl RNAse free water and elution buffer, respectively.

### Detection of *Borrelia* spp. by nested PCR, sequencing and phylogenetic analysis

Detection of *Borrelia* species in ticks was performed by a nested PCR using specific outer and inner primers for the 16S-23S intergenic spacer (16S-23S IGS) (Table 2) [32]. The amplification of a fragment of approximatively 1007 bp (size varies with the *Borrelia* species) in the first PCR and a fragment of 388–685 bp in the second PCR was performed as previously described [32], using GoTaq^®^ Flexi DNA Polymerase kit (Promega, Madison, USA). The PCR products from the nested PCR were separated on a 1.5% agarose gel stained with Roti^®^-GelStain Red (Carl Roth GmbH, Karlsruhe, Germany) and visualised with ChemiDoc™^M^ MP Imaging system (Bio-Rad Laboratories, Hercules, USA).

**Table 2:** Primer pairs used for detection or for the MLST of selected targets of *Borrelia* spp. in *I. ricinus* ticks

Pathogen	Target	Primer/Probe	Sequence (5′-3′)	References
*Borrelia* spp.	16S-23S rRNA	Bospp-IGS-F	GTATGTTTAGTGAGGGGGGTG	[32]
		Bospp-IGS-R	GGATCATAGCTCAGGTGGTTAG	
		Bospp-IGS-Fi	AGGGGGGTGAAGTCGTAACAAG	
		Bospp-IGS-Ri	GTCTGATAAACCTGAGGTCGGA	
*B. miyamotoi*	*nifS*	BmnifF31	GAAAAAGTAAACTCCCTCAGAAAGG	[35]
		BmnifR892	CAATGATGCCTGCAATATTTGGTG	
	*clpA*	BmclpAF1268	TTGATCTCTTAGATGATCTTGG	[35]
		BmclpAR2051	CAAACATAAACCTTTTCAGCCTTTAATA	
	*rlpB*	BmrplF18	ATTAAGACTTATARGCCAAAAAC	[35]
		BmrplR761	GGCTGNCCCCAAGGWGAT	
	*pyrG*	BmpyrF415	CTTYTAGTWATTGARATTGGTGGT	[35]
		BmpyrR1261	CAGCATCAAYTATRCCACAAAC	
	*recG*	BmrecF908	CTAGYATTCCTYTAATTGAGGC	[35]
		BmrecR1779	TTCRGTTAAAGGTTCCTTATAAAG	
	*clpX*	BmclpXF104	CTGTTGCYATTTGTTTTGAATGC(Y)TC	[35]
		BmclpXR1277	TAAAGTTCTTTTGCCCAAGG	
	*pepX*	BmpepXF361	AGAGAYTTAAGYTTAKCAGG	[35]
		BmpepXR1202	GTTTCTCTTAAAGAYTGCATTCC	
	*uvrA*	BmuvrF1435	GCTKAAATTTTTRATTGATGTTGGA	[35]
		BmuvrR2306	CARGGAACAAAAACATCRGGC	
	*rplB*^a^	BmrplF23	GACTTATAGGCCAAAAACTTC	[35]
		Bmrpl759R	GATACAGGATGACGACCACC	
	*pyrG*^a^	BmpyrF417	TTTAGTAATTGAGATTGGTGGTAC	[35]
		Bmpyr1252R	TATTCCACAAACATTACGAGC	
	*recG*^a^	BmrecF909	TAGCATTCCTTTAGTTGAGGC	[35]
		Bmrec1671R	CTCAGCATGCTCAACTACC	
	*clpX*^a^	BmclpF268	TTATCTGTTGCTGTTTATAATC	[35]
		BmclpX1155R	TTCAAACATAACATCTTTAAGTAATTCTTC	
	*pepX*^a^	BmpepF361	AGAGACTTAAATTTAGCAGGAGTTG	[35]
		Bmpep1187R	TGCATTCCCCACATTGGAGTTC	
	*uvrA*^a^	BmuvrF1437	TTAAATTTTTAATTGATGTTGGACT	[35]
		Bmuvr2147R	TCTGTAAAAAACCCAACATAAGTTGC	
TBEV	3′ non-coding region	F_TBE_1	GGGCGGTTCTTGTTCTCC	[36]
		R_TBE_1	ACACATCACCTCCTTGTCAGACT	
		TBE-probe-WT	TGAGCCACCATCACCCAGACACA	

^a^Sequencing primers used in the second round of amplification and for sequencing the PCR products

Positive samples were further processed for sequencing, first being purified with NucleoSEQ^®^ kit (Mackerey Nigel, Düren, Germany) following the manufacturer instructions, and then sequenced on the ABI PRISM^®^ 3130 sequencer in both directions using the inner primers (Table 2) [32], at the Institute of Diagnostic Virology, Friedrich-Loeffler-Institut, Germany.

The obtained nucleotide sequences were viewed and edited in Geneious 11.1.5 (https://www.geneious.com) and compared with data available in the GenBank database using BLASTn. Consensus *Borrelia* 16S-23S IGS sequences were deposited in the GenBank database under the Accession numbers MK945767-MK945887.

For performing the phylogenetic analysis, we have selected 20 representative *Borrelia* sequences and reference sequences from GenBank that had high rates of similarity, totalizing 39 nucleotide sequences. The tree was constructed by using the Maximum Likelihood method based on the Tamura 3-parameter model [33]. *Borrelia turicatae* (GenBank: MH620367) and *B. hermsii* (GenBank: KC883470) were used as the outgroup. The phylogenetic analysis was conducted in MEGA7 [34].

### Multilocus sequence typing of *Borrelia miyamotoi*

Multilocus sequence typing (MLST) was performed for seven samples that tested positive in nested PCR for 16S-23S IGS *B. miyamotoi* (GenBank: MK945785; MK945806; MK945817; MK945824; MK945861; MK945865; and MK945867). The selected seven samples were included in a PCR reaction using specific primer sets for the amplification of *nifS*, *clpA*, *rplB*, *pyrG*, *recG*, *clpX*, *pepX* and *uvrA* gene fragments for each sample, as previously described (Table 2) [35] with slight modifications. The amplification of targets was performed using GoTaq^®^ G2 Flexi DNA Polymerase kit (Promega). For *nifS* and *clpA* gene fragments, a touchdown PCR was undertaken by an initial denaturation step at 95 °C for 2 min, followed by a first set of cycles with annealing conditions at 58 °C decreasing with 1 °C until 50 °C for 30 s and extension at 72 °C for 60 s. Each reaction included 40 additional cycles at 95 °C for 30 s, 50 °C for 30 s and 72 °C for 60 s, followed by a final extension step at 72 °C for 5 min.

For the remaining six targets (*rplB*, *pyrG*, *recG*, *clpX*, *pepX* and *uvrA*), the touchdown PCR included a denaturation at 95 °C for 2 min, followed by a first set of cycles with an annealing temperature at 60 °C decreasing with 1 °C each cycle until 52 °C and an extension step at 72 °C for 60 s. Additionally, 40 cycles were run at 95 °C for 30 s, annealing at 50 °C, extension at 72 °C for 60 s and a final extension step at 72 °C for 5 min. PCR products from the reactions targeting *rplB*, *pyrG*, *recG*, *clpX*, *pepX* and *uvrA* were included in a nested PCR reaction, using sequencing primers (inner primers) for another round of amplification for 35 cycles, annealing temperature being set at 50 °C (Table 2) [35]. Sequences obtained for MLST analysis were aligned and compared to the available sequences from the MLST database by using the sequence query option (http://pubmlst.org/borrelia).

### Analysis of ticks for the detection of tick-borne encephalitis virus

RNA extracts were screened for tick-borne encephalitis virus (TBEV) by RT-qPCR targeting the 3′ non-coding region of the TBEV genome with specific primers and probe (Table 2) [36]. Taqman RT-qPCR reactions were performed in a final volume of 20 μl using the iTaq™ Universal Probes One Step Kit (BioRad Laboratory Inc., Munich, Germany). Each assay contained 10 μl Itaq universal probes reaction mix (2×), 2 μl of water, 400 nM of forward and reverse primers and 200 nM probe, 0.5 μl Iscript advanced reverse transcriptase and 5 μl of RNA. Each run included Langat virus RNA as positive control and water as negative control. Thermal cycling conditions of the reaction were as follows: 50 °C for 10 min, 95 °C for 5 min, 45 cycles at 95 °C for 15 s, then 60 °C for 1 min.

### Statistical analysis

The pathogens detected in the nymph pools were expressed as the percentage and minimum infection rate (MIR) calculated as the ratio of the number of positive pools to the total number of analyzed ticks, assuming that only one tick in each pool was positive. MIR was calculated according to site and *Borrelia* species detected in nymphal pools.

In order to compare the prevalence rates of *Borrelia* spp. in adult stages of ticks, infectious rates and MIR of different species, a t-test (two sample assuming unequal variances) was conducted using Microsoft Excel (2016) (Microsoft Corp., Redmont, USA). Confidence intervals (95% CI) were also calculated in Microsoft Excel 2016 for the infection rates and MIR of *Borrelia* spp. and for the detected *Borrelia* species in each of the developmental stages. *P*-values < 0.05 were considered significant.

## Results

In total, 2407 ticks were collected from April to October 2018 in 14 sites from Mecklenburg-Western Pomerania (Fig. 1), all identified as *I. ricinus*, of nymphal and adult stage only. Nymphs represented the dominant stage collected with 1936 individuals (80.4%), followed by females (239; 9.9%) and males (232; 9.6%) (Table 1). The highest number of ticks was collected from Kieshof, representing 58.4% (1406/2407) of the total ticks, followed by Weitenhagen (25.9%; 623/2407) and Mannhagen (6.2%; 149/2407) (Table 1). In these three locations, collection of ticks was performed monthly.

### *Borrelia* species detected in ticks

After performing nested PCR and sequencing the amplicons, the prevalence of *Borrelia* spp. in adult stages was identical: 12.1% (29/239 of females and 28/232 of males). MIR of 3.3% was registered in nymphs (64/1936) (Table 3).

**Table 3:** *Borrelia* species detected in adult and nymphal *I. ricinus* stage

Stage	*N*	*n*	No. of PCR positive, % (95% CI)				
			*Borrelia* spp.	*B. afzelii*	*B. garinii*	*B. valaisiana*	*B. miyamotoi*
Female	239	239	29/12.1 (8.0–16.3)	14/5.9 (2.9–8.9)	– –	8/3.4 (1.1–5.7)	7/2.9 (0.8–5.1)
Male	232	232	28/12.1 (7.8–16.3)	12/5.2 (2.3–8.0)	5/2.2 (0.3–4.0)	4/1.7 (0–3.4)	7/3.0 (0.8–5.2)
Nymph	1936	213^a^	64/3.3^b^ (2.5–4.1)	34/1.8^b^ (1.2–2.3)	1/0.1^b^ (0.0–0.2)	13/0.7^b^ (0.3–1.0)	16/0.8^b^ (0.4–1.2)

^a^Pooled samples

^b^MIR of pooled samples

*Abbreviation*: N, number of analyzed samples; n, number of samples/pools; –, no positive samples

Sequencing revealed the presence of four *Borrelia* species in analyzed ticks; three genospecies belonging to the *B. burgdorferi* (*s.l.*) group, *B. afzelii*, *B. garinii* and *B. valaisiana*, and one species of the relapsing fever group, *B. miyamotoi*. Out of these identified species, *B. afzelii* had the highest prevalence in adult ticks 5.5% (26/471). *Borrelia miyamotoi* was found in 3.0% (14/471) of adults, while *B. valaisiana* was detected in 2.5% (12/471) and *B. garinii* found in 1.1% (5/471) adult ticks (Table 3).

Amongst all four detected *Borrelia* species in adult ticks, *B. afzelii* had significantly higher infection rates than *B. garinii* (*P* *=* 0.0001) and *B. valaisiana* (*P* *=* 0.02) but not significantly different when compared to *B. miyamotoi* (*P* *=* 0.06). *Borrelia miyamotoi* showed a statistically significant higher prevalence in adults compared to *B. garinii* (*P* *=* 0.04) but not significantly different from *B. valaisiana* infection rate (*P* *=* 0.69). Infection rates of *Borrelia valaisiana* and *B. garinii* did not show significant differences (*P* *=* 0.09).

**Fig. 2:**
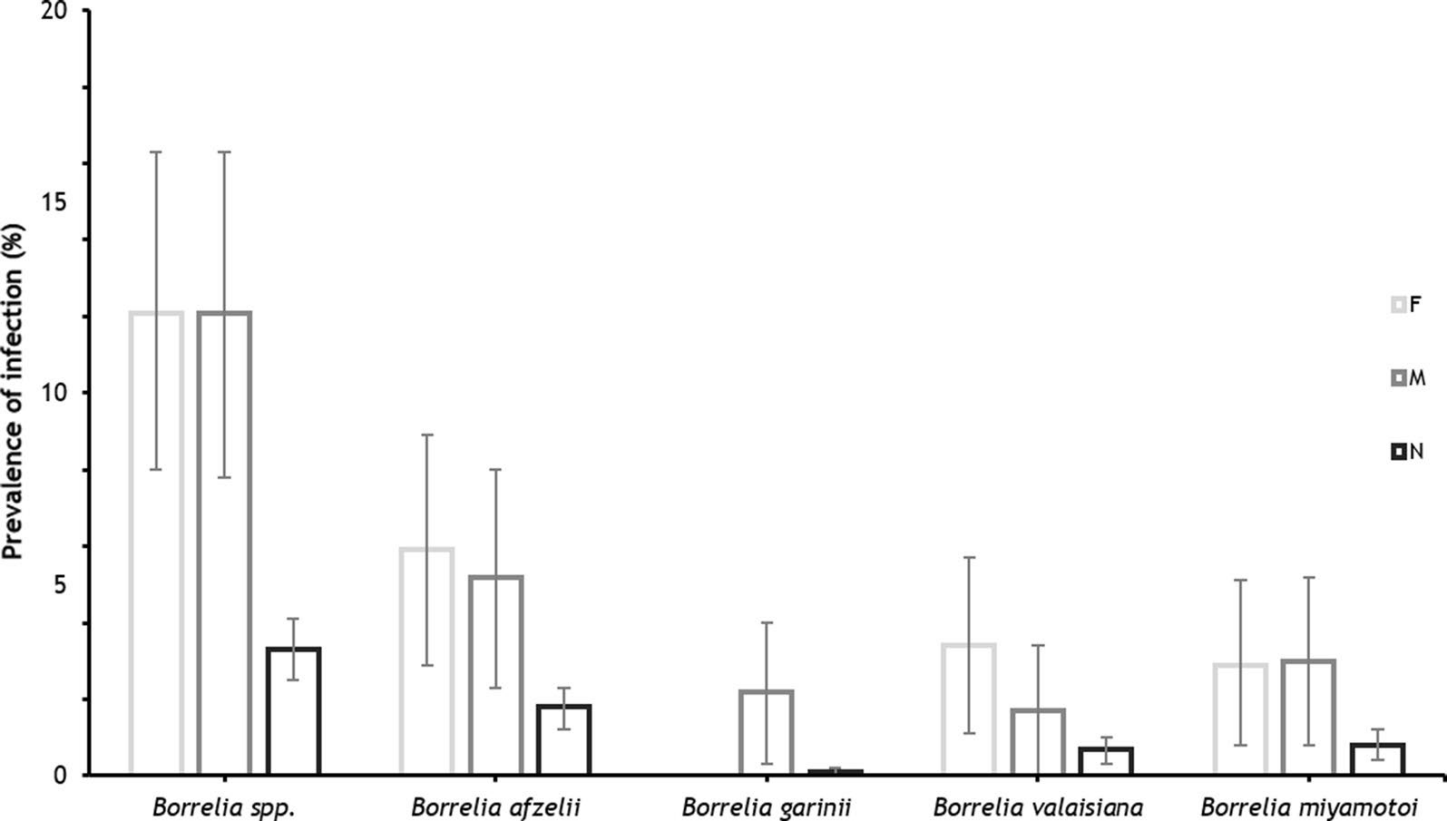
*Borrelia* species prevalence in *I. ricinus* adults and MIR in nymphs. *Abbreviations*: F, females; M, males; N, nymphs

*Borrelia afzelii* had similar infection rates in both female and male ticks, 5.9% (14/239) and 5.2% (12/232), respectively (*P* *=* 0.75). Nymphs showed a MIR for *B. afzelii* of 1.8% (34/1936) (Table 3, Fig. 2). *Borrelia miyamotoi* was detected in 2.9% (7/239) of *I. ricinus* females and 3.0% (7/232) of males (*P* *=* 0.96). MIR of *B. miyamotoi* in nymphs was 0.8% (16/1936) (Table 3, Fig. 2). Detection of *B. valaisiana* was confirmed in 3.4% (8/239) tick females and 1.7% (4/232) males, prevalence rates registering similar values (Table 3, Fig. 2) (*P* *=* 0.28). Pools of *I. ricinus* nymphs were also positive for *B. valaisiana*, MIR of 0.7% (13/1936) (Table 3, Fig. 2). All tested *I. ricinus* females were negative for *B. garinii*, while 2.2% of male ticks were positive (Table 3, Fig. 2). *Borrelia garinii* was found also in one pool of nymphs, MIR of 0.1% (1/1936) (Table 3, Fig. 2).

Considering the infection rates of different *Borrelia* species in each developmental stage, *I. ricinus* females had similar prevalence rate of *B. afzelii* when compared to *B. valaisiana* (5.9 *vs* 3.4%; *P* *=* 0.20) and *B. miyamotoi* (5.9 *vs* 2.9%; *P* *=* 0.13). In males, prevalence of *B. afzelii* was significantly higher than *B. valaisiana* (5.2 *vs* 1.7%; *P* *=* 0.04) but at similar rates with *B. miyamotoi* (5.2 *vs* 3.0%; *P* *=* 0.25) and *B. garinii* (5.2 *vs* 2.2%; *P* *=* 0.09) (Table 3, Fig. 2).

*Borrelia afzelii* MIR in nymphs was significantly higher compared to all other three *Borrelia* species detected (1.8 *vs* 0.8% *B. miyamotoi*, *P* *=* 0.01; *vs* 0.7% *B. valaisiana*, *P* *=* 0.002; *vs* 0.1% *B. garinii*, *P* *<* 0.0001). *Borrelia miyamotoi* and *B. valaisiana* had similar MIR (*P* *=* 0.57), and both species registered statistically higher MIR when compared to *B. garinii* (*P* *<* 0.0001 and *P* *=* 0.001, respectively) (Table 3, Fig. 2).

The highest number of positive ticks for *Borrelia* spp. was found in Kieshof (13.2% of adult ticks and MIR of 3.6% in nymphal pools), followed by Weitenhagen (13.1% of adult ticks and MIR of 2.6% in nymphal pools) (Table 4). The most prevalent species was *B. afzelii* for both sites: 5.5% of adult ticks and MIR of 2.0% for nymphs from Kieshof and 4.9% adults and MIR of 0.8% for nymphs from Weitenhagen. *Borrelia miyamotoi* was the second most prevalent species in both study sites (Kieshof: 4.0% of adult ticks and MIR of 0.7% in nymphs; Weitenhagen: 2.5% adult ticks and MIR of 1.2% in nymphal pools) (Table 4).

**Table 4:** Positive *Borrelia* spp. adult ticks and pool of nymphs from the study sites

Site	Location	No. of positive ticks per developmental stage														
		*Borrelia* spp.			*B. afzelii*			*B. garinii*			*B. valaisiana*			*B. miyamotoi*		
		F	M	N	F	M	N	F	M	N	F	M	N	F	M	N
1	Kieshofer	14	22	41	6	9	23	–	3	1	3	4	9	5	6	8
2	Ryck	–	–	1	–	–	–	–	–	–	–	–	–	–	–	1
3	Weitenhagen	13	3	13	6	–	4	–	2	–	5	–	3	2	1	6
4	Loitz	–	1	–	–	1	–	–	–	–	–	–	–	–	–	–
5	Carlsruhe	–	–	–	–	–	–	–	–	–	–	–	–	–	–	–
6	Tempel	–	1	–	–	1	–	–	–	–	–	–	–	–	–	–
7	Mannhagen	–	–	3	–	–	3	–	–	–	–	–	–	–	–	–
8	Riems	1	–	–	1	–	–	–	–	–	–	–	–	–	–	–
9	Ummanz	–	–	1	–	–	–	–	–	–	–	–	–	–	–	1
10	Ladebow	–	1	2	–	1	1	–	–	–	–	–	1	–	–	–
11	Horst	1	–	1	1	–	1	–	–	–	–	–	–	–	–	–
12	Thiesow	–	–	2	–	–	2	–	–	–	–	–	–	–	–	–
13	Wackerow	–	–	–	–	–	–	–	–	–	–	–	–	–	–	–
14	Annenhof	–	–	–	–	–	–	–	–	–	–	–	–	–	–	–
Total		29	28	64	14	12	34	–	5	1	8	4	13	7	7	16

*Abbreviations*: F, females; M, males; –, no positive samples

### Phylogenetic analysis of *Borrelia* species from Mecklenburg-Western Pomerania

**Fig. 3:**
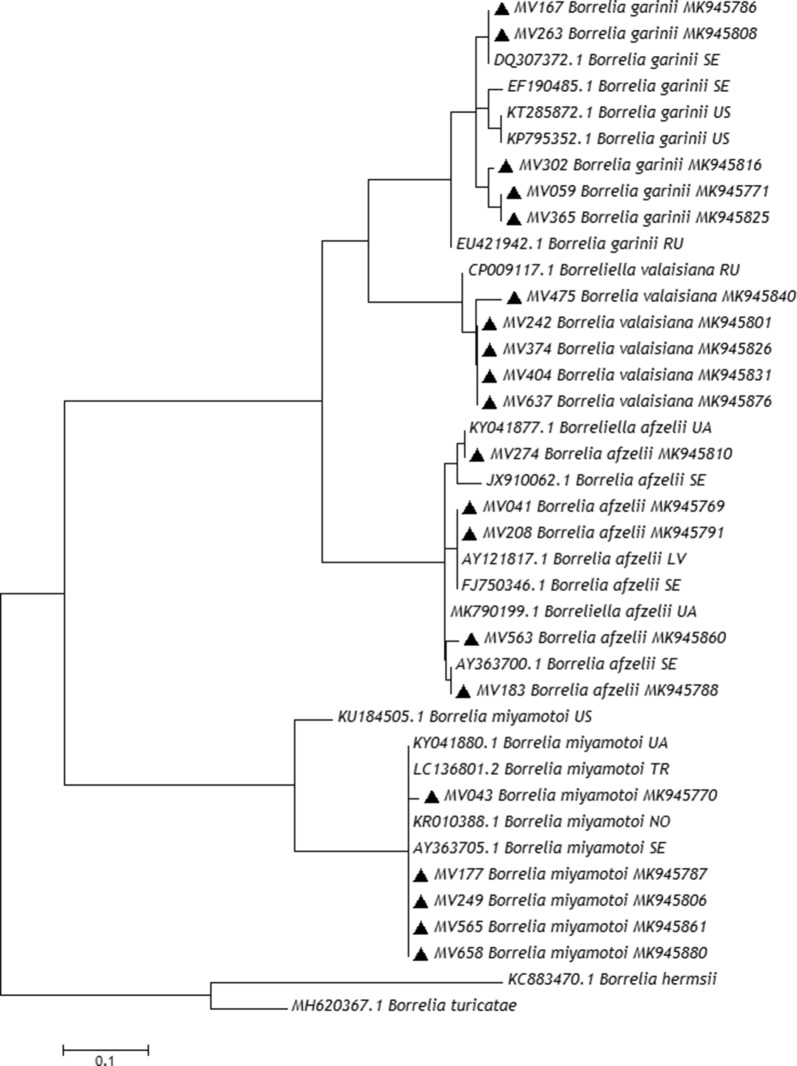
Phylogenetic analysis by Maximum Likelihood method. The evolutionary history of *Borrelia* spp. isolates, based on the 16S-23S IGS, was inferred by using the Maximum Likelihood method based on the Tamura 3-parameter model [33]. Initial tree(s) for the heuristic search were obtained automatically by applying Neighbor-Join and BioNJ algorithms to a matrix of pairwise distances estimated using the Maximum Composite Likelihood (MCL) approach, and then selecting the topology with superior log likelihood value. The tree is drawn to scale, with branch lengths measured in the number of substitutions per site. The analysis involved 39 nucleotide sequences. All positions containing gaps and missing data were eliminated. There were a total of 72 positions in the final dataset. Evolutionary analyses were conducted in MEGA7 [34]

Diversity of *Borrelia* species in north-eastern Germany based on the 16S-23S IGS included four different species. *Borrelia afzelii* sequences obtained in this study showed high similarity with isolate strains from Norway (GenBank: KY782011), Germany (GenBank: CP002933), Sweden (GenBank: FJ750344) and Ukraine (GenBank: MK790196). Sequences also clustered with strains isolated from Northern and north-eastern Europe (Fig. 3). Four out of six *B. garinii* isolates from this study were identical with strains isolated from *I. ricinus* larvae collected from the great tit *Parus major* in Sweden (GenBank: DQ307377 and DQ307377), while the other two were highly similar also to a strain from the same study in Sweden (GenBank: DQ307376). *Borrelia valaisiana* sequences obtained in this study, with sizes between 232–312 bp, matched only with the Russian strain Tom4006 (GenBank: CP009117) (Fig. 3). The majority of *B. miyamotoi* isolates from *I. ricinus* ticks collected from Mecklenburg-Western Pomerania were identical to isolate Z58 from Austria (GenBank: KP202177) and clustered with strains from Northern Europe and also from eastern European countries (Ukraine and Turkey) (Fig. 3).

### Multilocus sequence typing of *B. miyamotoi*

Seven samples identified as *B. miyamotoi* by 16S-23S IGS nested PCR were selected and analyzed by MLST, targeting eight highly conserved housekeeping genes (*nifS*, *clpA*, *rplB*, *pyrG*, *recG*, *clpX*, *pepX* and *uvrA*). Isolates were submitted to the *Borrelia* spp. MLST database (http://pubmlst.org/borrelia) (sample ID: 2654–2660) (complete batch of sequences is available in Additional file 1). All sequenced samples were *B. miyamotoi* and five of them (sample ID: 2654, 2655, 2656, 2657 and 2659) belonged to the sequence type (ST) 635. The other two samples (ID: 2658 and 2660) could not be framed in a ST due to poor quality sequences for *nifS*, but all alleles were distributed to the same ST 635 which corresponds to the EU_T01 isolate identified in 2014, in *I. ricinus* from Berlin, Germany.

### Detection of tick-borne encephalitis virus

The RT-qPCR performed on *I. ricinus* RNA did not identified positive samples for TBEV.

## Discussion

The objective of this study was to bring new insights regarding the circulation of *Borrelia* species and tick-borne encephalitis virus (TBEV) in *I. ricinus* ticks collected from vegetation in natural habitats from north-eastern Germany. *Ixodes ricinus* is an exophilic tick species with a wide distribution range in Europe known to feed on a great variety of hosts, records indicating more than 300 different vertebrate host species [37]. Due to these feeding preferences, *I. ricinus* can circulate various pathogens between host species, causing major health disorders of medical and veterinary importance. The most representative pathogens for which *I. ricinus* is the main vector in Europe are *B. burgdorferi* (*s.l.*), *B. miyamotoi*, TBEV, agents of the order Rickettsiales (*Rickettsia* spp. and *Anaplasma phagocytophilum*) and *Babesia* protozoans [37]. During our sampling, we managed to collect a relatively high number of *I. ricinus* ticks, nymphs being the most encountered stage in most of the study sites (Table 1). This finding is relevant for public health since nymphs represent the highest threat for humans compared to other tick stages due to their small size that enables them to go unnoticed during feeding corroborated with their marked anthropophily, abundance, and the ability to transmit a broad-range of pathogens [38, 39].

Detection of *B. burgdorferi* (*s.l.*) in *I. ricinus* ticks from Mecklenburg-Western-Pomerania confirms the role of this tick species as the main vector for Lyme disease genospecies. The overall prevalence of *B. burgdorferi* (*s.l.*) is lower compared to the infection rates registered in other studies from Germany [15, 16, 40–42]. However, a previous study reported similar infection rates in *I. ricinus* ticks (16.8% of females, 12.9% of males and 5.7% of nymphs) collected in 2003 from woodlands in Mecklenburg-Western Pomerania [43], potentially indicating a constant circulation of *B. burgdorferi* (*s.l.*) in the tick population from this region. Also, the minimum infection rate of nymphs reported in our study might actually be substituted by a higher infection rate, each pool potentially containing more than one positive nymph for *Borrelia* spp. As for the comparison between developmental stages, adult ticks had higher prevalence rates. This, in fact, can be explained by the life-cycle of hard ticks, with the adult stage having a greater chance to acquire the pathogens after feeding twice on two separate hosts, while for reaching nymphal stage only one feeding is necessary during the larval stage.

Sequencing revealed three genospecies of the *B. burgdorferi* (*s.l.*) complex, *B. afzelii* having the highest prevalence, followed by *B. valaisiana* and *B. garinii*. These results are in concordance with current knowledge indicating *B. afzelii* as the most common genospecies in Europe. In the event of clinical manifestations following *B. afzelii* infection, symptoms are represented by acrodermatitis chronica atrophicans and chronic skin disease [44]. Rodents are the reservoir hosts for *B. afzelii* in Europe (e.g. mice and voles) [45], representing important feeding hosts for immature stages of *I. ricinus.* Detection of *B. burgdorferi* (*s.l.*) in rodent populations from Germany with infection rates up to 49.1% confirmed the role of these small mammals as reservoir hosts [40, 42]. The collection sites for ticks in the current study are extensive forest areas that could contain high abundance of voles or other rodents acting as infected reservoir hosts for the local tick population.

*Borrelia valaisiana* was the second most prevalent genospecies in questing ticks from the studied area. Primary reservoir hosts for *B. valaisiana* and *Borrelia garinii* are ground-feeding birds (e.g. the common blackbird, *Turdus merula*, and the song thrush, *Turdus philomelos*) which feed in the habitats of ticks and become infested with these arthropods at higher rates [46]. In each year, over five million migratory birds and 27 species of water birds use habitats from Ruegen Island and the coast of Mecklenburg-West Pomerania as stopover sites in their migration between northern European countries and Central Europe and Africa [47]. This could mean that birds can also contribute to the long-distance distribution of tick-borne pathogens due to their role as reservoir hosts for infectious agents and also as hosts for immature stages of ticks.

Relapsing fever agent *Borrelia miyamotoi* was detected in 1.2% of the analysed ticks, this data being the first reported for Mecklenburg-Western Pomerania. In addition, the first available data regarding infection rates of *B. miyamotoi* in ticks from Northern Germany became available in 2018, when 2.1% of *I. ricinus* ticks collected from the city of Hanover were positive [16]. The prevalence of the current study is therefore in concordance with *B. miyamotoi* infectious rates found in *I. ricinus* ticks from Europe, ranging from 0.4% in Estonia to 3.5% in France and Germany [21, 48, 49]. The fact that this pathogen is present in Mecklenburg-Western Pomerania can have significance for clinicians since *B. miyamotoi* can cause febrile illness characterised by fever, nausea, fatigue, headache, chills, myalgia, arthralgia and meningoencephalitis [21].

The phylogenetic tree based on 16S-23S IGS shows that strains of *Borrelia* spp. found in the present study are closely related with isolates from Northern and north-eastern Europe (Fig. 3). This might indicate a circulation of these strains within this north-eastern region, most likely birds being involved in the dissemination in different regions of Northern Europe of the infected ticks and perhaps the *Borrelia* genospecies for which birds serve as reservoir hosts.

Multilocus sequence typing of eight housekeeping genes (*nifS*, *clpA, rplB*, *pyrG*, *recG*, *clpX*, *pepX* and *uvrA*) of *B. miyamotoi* isolates matched to ST 635 corresponding to the EU_T01 isolate identified in *I. ricinus* ticks from Berlin (https://pubmlst.org/bigsdb?page=info&db=pubmlst_borrelia_isolates&id=1279) which can indicate a low polymorphism between *B. miyamotoi* strains from the northern part of Germany. Performing MLST at a larger scale, by including an extensive number of isolates, will be useful to follow the subtle modifications between the strains and to study the phylogenetic relationships between *B. miyamotoi* strains isolated from Germany and Europe, this method having a high power of discrimination between strains [50].

Detection of TBEV in the tick samples analyzed in the present study was unsuccessful. In Germany, high endemic regions for TBEV are located in the southern part of the country (e.g. Bavaria and Baden-Wuerttemberg) but sporadic cases were also reported from the upper north regions of the country, recently including Mecklenburg-Western Pomerania [26]. Signs of TBEV circulation in this area were also shown in 2007 by detection of TBEV RNA in *I. ricinus* ticks [28] and identification of anti-TBEV antibodies in small ruminants [30], and wildlife [29]. Even though we did not detect TBEV RNA in the collected ticks, several factors could contribute to a future expansion of the virus foci in Mecklenburg-Western Pomerania. Climate change is one of the factors that was shown to influence the tick population in Northern Europe, increasing the duration of the vegetation period throughout the year and the humidity levels as well [51]. Also, changes in land use and movement of animal hosts could influence the abundance of tick populations in the region. Mecklenburg-Western Pomerania represents a popular destination for tourists that have many outdoor activities, raising the risk of exposure to tick bites in the area. Performing larger scale studies might contribute to an accurate estimation of TBEV infection rates in tick populations from Mecklenburg-Western Pomerania.

## Conclusions

The present study is adding new information regarding the distribution of *B. miyamotoi* in tick populations from Northern Germany which should be considered by medical clinicians when treating patients after a tick bite. The infection rate of *B. miyamotoi* in ticks was similar to other reports in tick populations from Europe suggesting that this relapsing fever agent might be well established in the local tick population and small mammal reservoir hosts. In addition, the detection of *B. afzelii*, *B. garinii* and *B. valaisiana* corroborating previous studies, suggests a constant circulation of *B. burgdorferi* (*s.l.*) in the tick population from this region. While in this study detection of tick-borne encephalitis virus was not possible, other reports showed signs of virus circulation in the area and extensive future studies should determine the natural foci of TBEV in Mecklenburg-Western Pomerania in order to reassess the risks for public health.

## Supplementary information

**Additional file 1.** Complete batch of *Borrelia miyamotoi* sequences corresponding to the following genes: *clpA*, *clpX*, *nifS*, *pepX*, *recG*, *pyrG*, *rplB*, *uvrA*.

## Data Availability

Consensus *Borrelia* 16S-23S IGS sequences were deposited in the GenBank database under the Accession numbers MK945767-MK945887.

## Abbreviations

MIR: Minimum infection rate
RT-qPCR: Reverse transcription quantitative polymerase chain reaction
BLAST: Basic local alignment search tool
IGS: Intergenic spacer
ST: Sequence type
CI: Confidence interval
MLST: Multilocus sequence typing
