# Supplementary material for: Borrelia miyamotoi and Borrelia burgdorferi (sensu lato) identification and survey of tick-borne encephalitis virus in ticks from north-eastern Germany
Source: Parasit Vectors. 2020 Feb 27;13:106. doi: 10.1186/s13071-020-3969-7 (PMC7045458; doi:10.1186/s13071-020-3969-7)
Supplement: Supplementary file 1 — Additional file 1. Complete batch of Borrelia miyamotoi sequences corresponding to the following genes: clpA, clpX, nifS, pepX, recG, pyrG, rplB, uvrA. [file 13071_2020_3969_MOESM1_ESM.docx]

**Additional file 1**. Complete batch of *Borrelia miyamotoi* sequences corresponding to the following genes: *clpA, clpX, nifS, pepX, recG, pyrG, rplB, uvrA*; Sequence type (ST) 635.

1. MLST for MV154 isolate

>MV154_*clpA*

GATGATATGAGAGACTTTGTTAAGTCTATGATTGGTTCTAATATTTTTAATTTTGATGATTATGATAATGAATTACTTATTAATCTGGAGAGTAAAATAAGAGAGCATATGATCATTGATGAAGAGCTTTTATCTGATTTGATATTAAACATAAAACTTTTAAGGATTAGATTTTTATTAAAAAATAATACTCTTGGTATTTTTATTTTAATGGGTTCCTCTGATATAAATAAGAGTAAACTTGCATCTATTTTATCAGAAGAGCTTAAGCTTCCTAAGTTTACTTTGGGAATGAGTGAGTATGGCGATTTTGATGGAATTAATAGATTAATAGGACCTGTATATGGAGCTGAGTTTTATGATGAACCTACTAAATTTTTTAAATTTTTAAGTAAAACTTCAAGCTCAATTATTTTCCTAGCAGATTTTGATAAATCTCCAAAGAGAGTCATAGATTTTTTTTTTGAAGGATTTAAGACTGGGAAACTTTTTGATAATTTTGGTAGAGATGTAAGTTTGTCAGATAGTATAATAATAATAGATATTAATGTGGATCACGGAGAACTTAATGGTATTGGATTTAAAAATGAGACAGTAAATAGCAGGAACCTACTAGAAAAGCGTTTTTCTAGTAGGTTCCTGGATTTAGTGGATCACATTTTTTTCTTTAGACCTGTAAATGAGAGTGATTTTAAAAGGGCTATTATCGAGGAGATGAATAATTTTGTTAAGATATTAAAGGCTGAAAAGGTTTATGTTT

>MV154_*clpX*

GTTTATAATCATTATAAAAGGATATTTAGGGGCAATAAGAACGAGAGTGGAGTTGAGCTTGAGAAATCTAATATATTGATTGTGGGTCCCACTGGAAGTGGTAAGACTTTGCTTGCAAAAAAGTTGGCAGCTGAGATGAATGTTCCATTTACAATTGCGGATGCTACAACGCTTACTGAAGCTGGATATGTAGGAGAAGATGTTGAGAACATTTTGCTTAAGCTCATTCATGCTGCGCATGGCGATGTAAGTTTCGCAGAACGAGGTATCATTTATATTGATGAAATAGATAAGATTGCAAAGAAAGGTGAAAATGTTTCAATTACAAGGGATGTTTCTGGAGAAGGTGTTCAACAATCTTTATTAAAGATAATTGAAGGAACTATTGCAAATGTTCCACCAAGGGGTGGGAGAAAACATCCTTATGAAGATACTATTGAAATTAATACACATAATATATTATTCATATGTGGTGGGGCTTTTGTAGGGCTTGAAAATATTATTAAGAAAAGAATTAATAAAAGCTCTATTGGATTTTCATCTGCTGGTAGGAAAGATGCCAAGGAAGATAATGTTTTGAAATATTTAGAGATGGAAGATTTGGTTAAATTTGGACTCATTCCAGAGTTTGTTGGTAGGCTTCCTGTGCATTCTTATCTTGAAAAGCTAGAAAAAGAAGATTTAATTAAAATATTAGTTGAACCTGAAAATTCTATTGTCAGACAATATTGTCATATGTTTAAGATGGATAATGTTGATCTGGTGTTTGAGAAGGATGCACTTGAAGTAATTGCAGAGGAAGCTATGATTAAAAATACAGGTGCAAGAGGTTTAAGATCTATTTTAGAAGAATTACTTAAAGATGTAG

>MV154_*nifS*

TACGCAAACTATAATGCAAATGTTCACAGAAGCGGACATGAACTTACAATACAATCCAGTTTAAAAGTAGAAGAAACAAGAAAAATTGTAAAAAGATTTATTAACGCAGAGTCTTGCAAAAACATAATCTTTAATTCTGGAACAACAGATGGAACAAATACAGTAACAAATTCATTGCTATTCTCAAAACTTTTAAAAGAAAACGATGAAATCATAGTAACAATGATAGAACACAATAGCAATTTGCTTCCTTGGATAAATATTGCCAAATTCTTAAACTTAAAGATTAAACTTGCAAAATTTAACGAAATGGGCATTATTCAACCAGAACAAATAAAAAACTTAATTACAGATAAAACAAAAATTATTGCTATATCTGGCATAAGCAATATACTAGGCACCATACAAGATTTAGAAGCAATTGGAAAAATTGCTAAAGATAACAAAATCATTCTATCTGTAGACGCAGCTCAAATGGCACCACATATGAACATAGATGTAAATAAAATAAATTGTGATTTTTTAGTATTCTCAGGGCACAAAATGCTTGCTCCAACAGGAATTGGAGTCTTATATATATCAAATAAAATCATAGATAAACTTAATAGCCCCAAACTAGGAGGAAATGCTATAGAAGATATTTTTATAAAAAATGGAGAGCTTAATTTTAAACCCCTTGAATCTCCAAATAAATTTGAATCGGGAACACCAAATATTGCAGGCATCATT

>MV154_*pepX*

TAAATTTAGCAGGAGTTGTATACTTTAATAAAGATGGAATAATTAACTCAGAACTAATAACAATTGAAAATATTGGAATTATACCAAATGTAGCTATTCACCTAAACCGACAAGTCAATGAAGGATTTGCATATGATGCTCATGAACACATTGTAATCATTACAAGTCTTCAAAAGAGCATTAAAGAAAAAATTCTAGAAAAACTGCAAATATCCAAAAAAGATTTTCTATCATGCGACTTAATATTCACAGCAGCTGAACCTGCCAAAATCATAGGCAGCGAGGGTGAATTTTTAGCATCCAAAAACCTTGACAATAAATCGGGATGTCACGCCATCATGAATGCATTTATTCACACTAATAACAATAAAAACAAAGTAATTGTATTTTTCGACAATGAAGAGATTGGGTCTTTAACTTCCAGAGGAGCTGATTCAAAACTATTAACAGAAGTCCTAGAAAGAATTGATCATGTTTTAAATTTAGGAAAAGAAGAACACTTAATAAAACTAAATAACTCATTCAACATTTCAATGGATGGTGCACATGGGGCTCATCCAGGTTACATAGACAAACATGACCCAAGCTATCAAATCAGTCTAGGTAAAGGAGTAACTATTAAAAGCAATGCTAACTTCAAATATGCAACAACAGCAAATAGCTGCGCAAAACTAAAATTACTAGCTATGAAAAACAATATTAAAATTCAAGAAATAATAATGAAAGCAAATACCAATACTGGAAGCACAATTGGTCCAATTTCAAATTCACAAACAGGTATTGAAACCATAGATATTGGAACTCCAATGTGGGA

>MV154_*pyrG*

ATGAGATTGGTGGTACTGTAGGGGATATGGAAAATATTTTGTTTATTGAGACAATGAGGCAGATAAGATATGAAATTGGGAGTGATAATATTGCTTTTATTCACTTGACTTATATTCCAAATCCTGTTGGGATAAATGAACAAAAGTCTAAACCTACACAACAGAGTGTAAAGACTTTAAATAAAGCTGGAATTTTTCCAGATTTAATTATTGCGAGAAATTCTCAACTTTTAACAAACCAGATAAGGCAAAAAATAGCAATGTTTTGTAATGTTGATGCTTCATCTATTATTGATAATGTTGATGTTTCTACTATTTATGAAATCCCTCTATCTTTTTATAAACAAGGCTTACATGAAATTTTGGGATCAAGATTAAAAATAAATGTTAAGCCCAAAGTGGATAGTCTTGATCGATTAGTTAATATAATAAAGAAAAATCTTGTATTTCCTAAGAAGGTAGTCAATATTGCTATTTGTGGTAAATATACTGAGCTTGGTGATTCTTATGCATCAATATTTGAGTCTTTAACGCATGTGTCTGCTAATTTAGATATGTTGGTTAAGACAACTGTAATTGATAGTACTAATTTTAATGAGGAGATGTTGAAGAATATAGATGGTATAGTAGTGCCTGGTGGTTTTGGTGGTAGAGGATATGAAGGTAAAATTCGTGCAATTAAATATGCTCGTGAAAATAATATTCCTTTTCTTGGTATTTGTCTTGGTATGCAACTTGCAGTAATTGAATTCGCTCGTAATGTT

>MV154_*recG*

TAGCATTCCTTTAGTTGAGGCTGGATATCAAGTTGCATTAATGGTGCCTACTGATCTTTTGGCTAAGCAACATTATAGTAATTTGTCAAGCATATTAAAAGATTTTAATGTTTCTATAGCTCTTTTAACTGGTAGTTTAAAGAAGAGAGATAGGGACGATGTTTTGAAAAAACTTCAGAGTGGGATTTATAGTTTAGTAATTGGGACTCATGCTATTTTTGATCAAGGAACAAAATTTAAAAAATTAGCTTATATTATTATTGATGAACAGCATAAATTTGGTGTTGAACAAAGAGAGGCCCTTAAAAATAAGGGAGAAGGAGTTGATGTTCTTTTAATGTCAGCGACTCCTATTCCTAGGAGCTTAGCTTTGACTCTTTTTGGTGATCTTCAGGTATCTTTAATTAAGAAAGGTCCTGCAGGTCGAATACCCGTTACTACTTATTTAGCGAAGCATGGAAATGAAAAAAAAGTGTATGAGTTTTTAAAAAATGAACTTGCAAAAGGGCATCAAGTTTATTTTGTTTATCCCCTAATATCATCTTCACAGAAGTTTGATTTAAAAGATGCTACTAGTATGTGTTTAACGCTTAAGAATATTTTTGTTGAGTATTCTGTTGCTATGATTCATTCTAAGCTTGAGTCTAATATCAAAGAAGAAATTATGCAGGATTTTTATTTGAGGAAAATAGATATTTTAGTTGCAACAAG

>MV154_*rplB*

CTTTAACTAAGGGGAAGGTATCTAGGGCTGGAAGAGATTCTTCTGGAAGAATTAGTGTTAGAAGAAGGGGTGGAGGACATAAAAGACGGTATAGGGAAATTGACTTTGGAAGAAGAGATAAGTTTGATATACCTGCTCGAGTTGTGTCTATTGAATATGATCCAAATAGAAGTTCTAATATAGCTTTACTTGTTTATAAGGATGGAGATAAAAGATATATTATTGCTCCTAAAGGCATTAAAGTAGGTGATGTTTTACAAAGTGGTCCAAAGGCTCCAATAAGGGTTGGTAATTCTTTGCCGCTTGAAAACATTCCTGTTGGTAAGGCAGTACATAACATTGAACTTAATGTTGGAAGAGGTGGACAGCTTGTAAGAGGTGCTGGGGGATATGCCATGGTGCTTGCTTCTGAGGGAAATTATGTAACAGTTAAGCTTCCATCAGGTGAGGTTCGCATGGTTTTTAAGAAATGCATGGCAACTCTTGGAGAAGTTGGGAATGAAAATTATATTAATGTTTCTATTGGTAAGGCTGGTAAGAGTAGATGGCTTGGTAAAAGGCCTAAGGTAAGAGGTGTTGCCATGAATCCTGTCGATCACCCACATGGAGGTGGTGAGGGTAAGACTTCTGGTGGTCGTCATC

>MV154_*uvrA*

TTTTAATTGATGTTGGACTTTCTTATTTATATTTAGATAGGATGTCTGGTACCCTTTCAGGGGGGGAGGCTCAACGGAATTAGGCTTGCTACTCAAATAGGTTCAGCTCTTGCTGGAGTTCTTTATATACTTGATGAACCTAGTATTGGATTGCATCAAAGAGATAATGAAAAGTTAATAAGTACTCTTATTAGTTTGAAAGAACTTGGCAATACAGTAATTGTTGTAGAGCATGATGAGCAAACTTTGCGTACTGCTGATTATATTGTCGATGTTGGGCCTGGAGCTGGTATTCATGGTGGAGAGATAGTTGCTCAGGGAACTTTAGCTGATATTTTGAATAATGAAAATAGTTTGACTGGAAAGTATTTAAGTGGTCAGCTTAAAATAGAAGTCCCAAAAACAAGGCGTAAGAAGGGCAAAGAAGAAATTTTGCTTTTAAATGCTAATAAAAACAATTTAAAGAATATTAATGTTTGCATTCCTTTGGGAATTTTTACTGTAATAACGGGAGTTTCTGGTAGTGGTAAAAGTACTCTTTTAAATGAAGTATTATATCCTGCTCTTGATAGTAGGTTGAAATTAAATACAAGTTATTTTGATGGGTTTGAGAATATCATTGGGTATGAACAAATTGACAAAATTATTCAGATAAATCAAAAGCCAATAGGCAAGACACCAAGATCAAATCCTGCAACTTATGTTGGGTTATT

2. MLST for MV249 isolate

>MV249_*clpA*

GATGATATGAGAGACTTTGTTAAGTCTATGATTGGTTCTAATATTTTTAATTTTGATGATTATGATAATGAATTACTTATTAATCTGGAGAGTAAAATAAGAGAGCATATGATCATTGATGAAGAGCTTTTATCTGATTTGATATTAAACATAAAACTTTTAAGGATTAGATTTTTATTAAAAAATAATACTCTTGGTATTTTTATTTTAATGGGTTCCTCTGATATAAATAAGAGTAAACTTGCATCTATTTTATCAGAAGAGCTTAAGCTTCCTAAGTTTACTTTGGGAATGAGTGAGTATGGCGATTTTGATGGAATTAATAGATTAATAGGACCTGTATATGGAGCTGAGTTTTATGATGAACCTACTAAATTTTTTAAATTTTTAAGTAAAACTTCAAGCTCAATTATTTTCCTAGCAGATTTTGATAAATCTCCAAAGAGAGTCATAGATTTTTTTTTTGAAGGATTTAAGACTGGGAAACTTTTTGATAATTTTGGTAGAGATGTAAGTTTGTCAGATAGTATAATAATAATAGATATTAATGTGGATCACGGAGAACTTAATGGTATTGGATTTAAAAATGAGACAGTAAATAGCAGGAACCTACTAGAAAAGCGTTTTTCTAGTAGGTTCCTGGATTTAGTGGATCACATTTTTTTCTTTAGACCTGTAAATGAGAGTGATTTTAAAAGGGCTATTATCGAGGAGATGAATAATTTTGTTAAGATATTAAAGGCTGAAAAGGTTTATGTTT

>MV249_*clpX*

AAAGGATATTTAGGGGCAATAAGAACGAAAGTGGAGTTGAGCTTGAGAAATCTAATATATTGATTGTGGGTCCCACTGGAAGTGGTAAGACTTTGCTTGCAAAAAAGTTGGCAGCTGAGATGAATGTTCCATTTACAATTGCGGATGCTACAACGCTTACTGAAGCTGGATATGTAGGAGAAGATGTTGAGAACATTTTGCTTAAGCTCATTCATGCTGCGCATGGCGATGTAAGTTTCGCAGAACGAGGTATCATTTATATTGATGAAATAGATAAGATTGCAAAGAAAGGTGAAAATGTTTCAATTACAAGGGATGTTTCTGGAGAAGGTGTTCAACAATCTTTATTAAAGATAATTGAAGGAACTATTGCAAATGTTCCACCAAGGGGTGGGAGAAAACATCCTTATGAAGATACTATTGAAATTAATACACATAATATATTATTCATATGTGGTGGGGCTTTTGTAGGGCTTGAAAATATTATTAAGAAAAGAATTAATAAAAGCTCTATTGGATTTTCATCTGCTGGTAGGAAAGATGCCAAGGAAGATAATGTTTTGAAATATTTAGAGATGGAAGATTTGGTTAAATTTGGACTCATTCCAGAGTTTGTTGGTAGGCTTCCTGTGCATTCTTATCTTGAAAAGCTAGAAAAAGAAGATTTAATTAAAATATTAGTTGAACCTGAAAATTCTATTGTCAGACAATATTGTCATATGTTTAAGATGGATAATGTTGATCTGGTGTTTGAGAAGGATGCACTTGAAGTAATTGCAGAGGAAGCTATGATTAAAAATACAGGTGCAAGAGGTTTAAGATCTATTTTAGAAGAATTACTTAAAGATGTAGT

>MV249_*nifS*

AATACTACGCAAACTATAATGCAAATGTTCACAGAAGCGGACATGAACTTACAATACAATCCAGTTTAAAAGTAGAAGAAACAAGAAAAATTGTAAAAAGATTTATTAACGCAGAGTCTTGCAAAAACATAATCTTTAATTCTGGAACAACAGATGGAACAAATACAGTAACAAATTCATTGCTATTCTCAAAACTTTTAAAAGAAAACGATGAAATCATAGTAACAATGATAGAACACAATAGCAATTTGCTTCCTTGGATAAATATTGCCAAATTCTTAAACTTAAAGATTAAACTTGCAAAATTTAACGAAATGGGCATTATTCAACCAGAACAAATAAAAAACTTAATTACAGATAAAACAAAAATTATTGCTATATCTGGCATAAGCAATATACTAGGCACCATACAAGATTTAGAAGCAATTGGAAAAATTGCTAAAGATAACAAAATCATTCTATCTGTAGACGCAGCTCAAATGGCACCACATATGAACATAGATGTAAATAAAATAAATTGTGATTTTTTAGTATTCTCAGGGCACAAAATGCTTGCTCCAACAGGAATTGGAGTCTTATATATATCAAATAAAATCATAGATAAACTTAATAGCCCCAAACTAGGAGGAAATGCTATAGAAGATATTTTTATAAAAAATGGAGAGCTTAATTTTAAACCCCTTGAATCTCCAAATAAATTTGAATCGGGAACACCAAATATTGCAGGCATCATT

>MV249_*pepX*

TAAATTTAGCAGGAGTTGTATACTTTAATAAAGATGGAATAATTAACTCAGAACTAATAACAATTGAAAATATTGGAATTATACCAAATGTAGCTATTCACCTAAACCGACAAGTCAATGAAGGATTTGCATATGATGCTCATGAACACATTGTAATCATTACAAGTCTTCAAAAGAGCATTAAAGAAAAAATTCTAGAAAAACTGCAAATATCCAAAAAAGATTTTCTATCATGCGACTTAATATTCACAGCAGCTGAACCTGCCAAAATCATAGGCAGCGAGGGTGAATTTTTAGCATCCAAAAACCTTGACAATAAATCGGGATGTCACGCCATCATGAATGCATTTATTCACACTAATAACAATAAAAACAAAGTAATTGTATTTTTCGACAATGAAGAGATTGGGTCTTTAACTTCCAGAGGAGCTGATTCAAAACTATTAACAGAAGTCCTAGAAAGAATTGATCATGTTTTAAATTTAGGAAAAGAAGAACACTTAATAAAACTAAATAACTCATTCAACATTTCAATGGATGGTGCACATGGGGCTCATCCAGGTTACATAGACAAACATGACCCAAGCTATCAAATCAGTCTAGGTAAAGGAGTAACTATTAAAAGCAATGCTAACTTCAAATATGCAACAACAGCAAATAGCTGCGCAAAACTAAAATTACTAGCTATGAAAAACAATATTAAAATTCAAGAAATAATAATGAAAGCAAATACCAATACTGGAAGCACAATTGGTCCAATTTCAAATTCACAAACAGGTATTGAAACCATAGATATTGGAACTCCAATGTGGGA

>MV249_*pyrG*

ATGAGATTGGTGGTACTGTAGGGGATATGGAAAATATTTTGTTTATTGAGACAATGAGGCAGATAAGATATGAAATTGGGAGTGATAATATTGCTTTTATTCACTTGACTTATATTCCAAATCCTGTTGGGATAAATGAACAAAAGTCTAAACCTACACAACAGAGTGTAAAGACTTTAAATAAAGCTGGAATTTTTCCAGATTTAATTATTGCGAGAAATTCTCAACTTTTAACAAACCAGATAAGGCAAAAAATAGCAATGTTTTGTAATGTTGATGCTTCATCTATTATTGATAATGTTGATGTTTCTACTATTTATGAAATCCCTCTATCTTTTTATAAACAAGGCTTACATGAAATTTTGGGATCAAGATTAAAAATAAATGTTAAGCCCAAAGTGGATAGTCTTGATCGATTAGTTAATATAATAAAGAAAAATCTTGTATTTCCTAAGAAGGTAGTCAATATTGCTATTTGTGGTAAATATACTGAGCTTGGTGATTCTTATGCATCAATATTTGAGTCTTTAACGCATGTGTCTGCTAATTTAGATATGTTGGTTAAGACAACTGTAATTGATAGTACTAATTTTAATGAGGAGATGTTGAAGAATATAGATGGTATAGTAGTGCCTGGTGGTTTTGGTGGTAGAGGATATGAAGGTAAAATTCGTGCAATTAAATATGCTCGTGAAAATAATATTCCTTTTCTTGGTATTTGTCTTGGTATGCAACTTGCAGTAATTGAATTCGCTCGTAATGTTTGTGGGAA

>MV249_*recG*

TAGCATTCCTTTAGTTGAGGCTGGATATCAAGTTGCATTAATGGTGCCTACTGATCTTTTGGCTAAGCAACATTATAGTAATTTGTCAAGCATATTAAAAGATTTTAATGTTTCTATAGCTCTTTTAACTGGTAGTTTAAAGAAGAGAGATAGGGACGATGTTTTGAAAAAACTTCAGAGTGGGATTTATAGTTTAGTAATTGGGACTCATGCTATTTTTGATCAAGGAACAAAATTTAAAAAATTAGCTTATATTATTATTGATGAACAGCATAAATTTGGTGTTGAACAAAGAGAGGCCCTTAAAAATAAGGGAGAAGGAGTTGATGTTCTTTTAATGTCAGCGACTCCTATTCCTAGGAGCTTAGCTTTGACTCTTTTTGGTGATCTTCAGGTATCTTTAATTAAGAAAGGTCCTGCAGGTCGAATACCCGTTACTACTTATTTAGCGAAGCATGGAAATGAAAAAAAAGTGTATGAGTTTTTAAAAAATGAACTTGCAAAAGGGCATCAAGTTTATTTTGTTTATCCCCTAATATCATCTTCACAGAAGTTTGATTTAAAAGATGCTACTAGTATGTGTTTAACGCTTAAGAATATTTTTGTTGAGTATTCTGTTGCTATGATTCATTCTAAGCTTGAGTCTAATATCAAAGAAGAAATTATGCAGGATTTTTATTTGAGGAAAATAGATATTTTAGTTGCAACAAGTGTT

>MV249_*rplB*

TAGGGCTGGAAGAGATTCTTCTGGAAGAATTAGTGTTAGAAGAAGGGGTGGAGGACATAAAAGACGGTATAGGGAAATTGACTTTGGAAGAAGAGATAAGTTTGATATACCTGCTCGAGTTGTGTCTATTGAATATGATCCAAATAGAAGTTCTAATATAGCTTTACTTGTTTATAAGGATGGAGATAAAAGATATATTATTGCTCCTAAAGGCATTAAAGTAGGTGATGTTTTACAAAGTGGTCCAAAGGCTCCAATAAGGGTTGGTAATTCTTTGCCGCTTGAAAACATTCCTGTTGGTAAGGCAGTACATAACATTGAACTTAATGTTGGAAGAGGTGGACAGCTTGTAAGAGGTGCTGGGGGATATGCCATGGTGCTTGCTTCTGAGGGAAATTATGTAACAGTTAAGCTTCCATCAGGTGAGGTTCGCATGGTTTTTAAGAAATGCATGGCAACTCTTGGAGAAGTTGGGAATGAAAATTATATTAATGTTTCTATTGGTAAGGCTGGTAAGAGTAGATGGCTTGGTAAAAGGCCTAAGGTAAGAGGTGTTGCCATGAATCCTGTCGATCACCCACATGGAGGTGGTGAGGGTAAGACTTCTGGTGGTCGTCATC

>MV249_*uvrA*

TTTTAATTGATGTTGGACTTTCTTATTTATATTTAGATAGGATGTCTGGTACCCTTTCAGGGGGGGAGGCTCAACGAATTAGGCTTGCTACTCAAATAGGTTCAGCTCTTGCTGGAGTTCTTTATATACTTGATGAACCTAGTATTGGATTGCATCAAAGAGATAATGAAAAGTTAATAAGTACTCTTATTAGTTTGAAAGAACTTGGCAATACAGTAATTGTTGTAGAGCATGATGAGCAAACTTTGCGTACTGCTGATTATATTGTCGATGTTGGGCCTGGAGCTGGTATTCATGGTGGAGAGATAGTTGCTCAGGGAACTTTAGCTGATATTTTGAATAATGAAAATAGTTTGACTGGAAAGTATTTAAGTGGTCAGCTTAAAATAGAAGTCCCAAAAACAAGGCGTAAGAAGGGCAAAGAAGAAATTTTGCTTTTAAATGCTAATAAAAACAATTTAAAGAATATTAATGTTTGCATTCCTTTGGGAATTTTTACTGTAATAACGGGAGTTTCTGGTAGTGGTAAAAGTACTCTTTTAAATGAAGTATTATATCCTGCTCTTGATAGTAGGTTGAAATTAAATACAAGTTATTTTGATGGGTTTGAGAATATCATTGGGTATGAACAAATTGACAAAATTATTCAGATAAATCAAAAGCCAATAGGCAAGACACCAAGATCAAATCCTGCAACTTATGTTGGGTTATT

3. MLST for MV307 isolate

>MV307_*clpA*

ATATTTTTAATTTTGATGATTATGATAATGAATTACTTATTAATCTGGAGAGTAAAATAAGAGAGCATATGATCATTGATGAAGAGCTTTTATCTGATTTGATATTAAACATAAAACTTTTAAGGATTAGATTTTTATTAAAAAATAATACTCTTGGTATTTTTATTTTAATGGGTTCCTCTGATATAAATAAGAGTAAACTTGCATCTATTTTATCAGAAGAGCTTAAGCTTCCTAAGTTTACTTTGGGAATGAGTGAGTATGGCGATTTTGATGGAATTAATAGATTAATAGGACCTGTATATGGAGCTGAGTTTTATGATGAACCTACTAAATTTTTTAAATTTTTAAGTAAAACTTCAAGCTCAATTATTTTCCTAGCAGATTTTGATAAATCTCCAAAGAGAGTCATAGATTTTTTTTTTGAAGGATTTAAGACTGGGAAACTTTTTGATAATTTTGGTAGAGATGTAAGTTTGTCAGATAGTATAATAATAATAGATATTAATGTGGATCACGGAGAACTTAATGGTATTGGATTTAAAAATGAGACAGTAAATAGCAGGAACCTACTAGAAAAGCGTTTTTCTAGTAGGTTCCTGGATTTAGTGGATCACATTTTTTTCTTTAGACCTGTAAATGAGAGTGATTTTAAAAGGGCTATTATCGAGGAGATGAATAATTTTGTTAAGATATTAAAGGCTGAAAAGGTTTATGTT

>MV307_*clpX*

ATCATTATAAAAGGATATTTAGGGGCAATAAGAACGAAAGTGGAGTTGAGCTTGAGAAATCTAATATATTGATTGTGGGTCCCACTGGAAGTGGTAAGACTTTGCTTGCAAAAAAGTTGGCAGCTGAGATGAATGTTCCATTTACAATTGCGGATGCTACAACGCTTACTGAAGCTGGATATGTAGGAGAAGATGTTGAGAACATTTTGCTTAAGCTCATTCATGCTGCGCATGGCGATGTAAGTTTCGCAGAACGAGGTATCATTTATATTGATGAAATAGATAAGATTGCAAAGAAAGGTGAAAATGTTTCAATTACAAGGGATGTTTCTGGAGAAGGTGTTCAACAATCTTTATTAAAGATAATTGAAGGAACTATTGCAAATGTTCCACCAAGGGGTGGGAGAAAACATCCTTATGAAGATACTATTGAAATTAATACACATAATATATTATTCATATGTGGTGGGGCTTTTGTAGGGCTTGAAAATATTATTAAGAAAAGAATTAATAAAAGCTCTATTGGATTTTCATCTGCTGGTAGGAAAGATGCCAAGGAAGATAATGTTTTGAAATATTTAGAGATGGAAGATTTGGTTAAATTTGGACTCATTCCAGAGTTTGTTGGTAGGCTTCCTGTGCATTCTTATCTTGAAAAGCTAGAAAAAGAAGATTTAATTAAAATATTAGTTGAACCTGAAAATTCTATTGTCAGACAATATTGTCATATGTTTAAGATGGATAATGTTGATCTGGTGTTTGAGAAGGATGCACTTGAAGTAATTGCAGAGGAAGCTATGATTAAAAATACAGGTGCAAGAGGTTTAAGATCTATTTTAGAAGAATTACTTAAAGATGTAG

>MV307_*nifS*

TCACAGAAGCGGACATGAACTTACAATACAATCCAGTTTAAAAGTAGAAGAAACAAGAAAAATTGTAAAAAGATTTATTAACGCAGAGTCTTGCAAAAACATAATCTTTAATTCTGGAACAACAGATGGAACAAATACAGTAACAAATTCATTGCTATTCTCAAAACTTTTAAAAGAAAACGATGAAATCATAGTAACAATGATAGAACACAATAGCAATTTGCTTCCTTGGATAAATATTGCCAAATTCTTAAACTTAAAGATTAAACTTGCAAAATTTAACGAAATGGGCATTATTCAACCAGAACAAATAAAAAACTTAATTACAGATAAAACAAAAATTATTGCTATATCTGGCATAAGCAATATACTAGGCACCATACAAGATTTAGAAGCAATTGGAAAAATTGCTAAAGATAACAAAATCATTCTATCTGTAGACGCAGCTCAAATGGCACCACATATGAACATAGATGTAAATAAAATAAATTGTGATTTTTTAGTATTCTCAGGGCACAAAATGCTTGCTCCAACAGGAATTGGAGTCTTATATATATCAAATAAAATCATAGATAAACTTAATAGCCCCAAACTAGGAGGAAATGCTATAGAAGATATTTTTATAAAAAATGGAGAGCTTAATTTTAAACCCCTTGAATCTCCAAATAAATTTGAAATCGGGGAACACC

>MV307_*pepX*

TTAGAGAACTAAATTTAGCAGGAGTTGTATACTTTAATAAAGATGGAATAATTAACTCAGAACTAATAACAATTGAAAATATTGGAATTATACCAAATGTAGCTATTCACCTAAACCGACAAGTCAATGAAGGATTTGCATATGATGCTCATGAACACATTGTAATCATTACAAGTCTTCAAAAGAGCATTAAAGAAAAAATTCTAGAAAAACTGCAAATATCCAAAAAAGATTTTCTATCATGCGACTTAATATTCACAGCAGCTGAACCTGCCAAAATCATAGGCAGCGAGGGTGAATTTTTAGCATCCAAAAACCTTGACAATAAATCGGGATGTCACGCCATCATGAATGCATTTATTCACACTAATAACAATAAAAACAAAGTAATTGTATTTTTCGACAATGAAGAGATTGGGTCTTTAACTTCCAGAGGAGCTGATTCAAAACTATTAACAGAAGTCCTAGAAAGAATTGATCATGTTTTAAATTTAGGAAAAGAAGAACACTTAATAAAACTAAATAACTCATTCAACATTTCAATGGATGGTGCACATGGGGCTCATCCAGGTTACATAGACAAACATGACCCAAGCTATCAAATCAGTCTAGGTAAAGGAGTAACTATTAAAAGCAATGCTAACTTCAAATATGCAACAACAGCAAATAGCTGCGCAAAACTAAAATTACTAGCTATGAAAAACAATATTAAAATTCAAGAAATAATAATGAAAGCAAATACCAATACTGGAAGCACAATTGGTCCAATTTCAAATTCACAAACAGGTATTGAAACCATAGATATTGGAACTCCAATGTGGGAAA

>MV307_*pyrG*

ATTGAGATTGGTGGTACTGTAGGGGATATGGAAAATATTTTGTTTATTGAGACAATGAGGCAGATAAGATATGAAATTGGGAGTGATAATATTGCTTTTATTCACTTGACTTATATTCCAAATCCTGTTGGGATAAATGAACAAAAGTCTAAACCTACACAACAGAGTGTAAAGACTTTAAATAAAGCTGGAATTTTTCCAGATTTAATTATTGCGAGAAATTCTCAACTTTTAACAAACCAGATAAGGCAAAAAATAGCAATGTTTTGTAATGTTGATGCTTCATCTATTATTGATAATGTTGATGTTTCTACTATTTATGAAATCCCTCTATCTTTTTATAAACAAGGCTTACATGAAATTTTGGGATCAAGATTAAAAATAAATGTTAAGCCCAAAGTGGATAGTCTTGATCGATTAGTTAATATAATAAAGAAAAATCTTGTATTTCCTAAGAAGGTAGTCAATATTGCTATTTGTGGTAAATATACTGAGCTTGGTGATTCTTATGCATCAATATTTGAGTCTTTAACGCATGTGTCTGCTAATTTAGATATGTTGGTTAAGACAACTGTAATTGATAGTACTAATTTTAATGAGGAGATGTTGAAGAATATAGATGGTATAGTAGTGCCTGGTGGTTTTGGTGGTAGAGGATATGAAGGTAAAATTCGTGCAATTAAATATGCTCGTGAAAATAATATTCCTTTTCTTGGTATTTGTCTTGGTATGCAACTTGCAGTAATTGAATTCGCTCGTAATGTT

>MV307_*recG*

AGTTGAGGCTGGATATCAAGTTGCATTAATGGTGCCTACTGATCTTTTGGCTAAGCAACATTATAGTAATTTGTCAAGCATATTAAAAGATTTTAATGTTTCTATAGCTCTTTTAACTGGTAGTTTAAAGAAGAGAGATAGGGACGATGTTTTGAAAAAACTTCAGAGTGGGATTTATAGTTTAGTAATTGGGACTCATGCTATTTTTGATCAAGGAACAAAATTTAAAAAATTAGCTTATATTATTATTGATGAACAGCATAAATTTGGTGTTGAACAAAGAGAGGCCCTTAAAAATAAGGGAGAAGGAGTTGATGTTCTTTTAATGTCAGCGACTCCTATTCCTAGGAGCTTAGCTTTGACTCTTTTTGGTGATCTTCAGGTATCTTTAATTAAGAAAGGTCCTGCAGGTCGAATACCCGTTACTACTTATTTAGCGAAGCATGGAAATGAAAAAAAAGTGTATGAGTTTTTAAAAAATGAACTTGCAAAAGGGCATCAAGTTTATTTTGTTTATCCCCTAATATCATCTTCACAGAAGTTTGATTTAAAAGATGCTACTAGTATGTGTTTAACGCTTAAGAATATTTTTGTTGAGTATTCTGTTGCTATGATTCATTCTAAGCTTGAGTCTAATATCAAAGAAGAAATTATGCAGGATTTTTATTTGAGGAAAATAGATATTTTAGTTGCAACAAGTGTTATTGAAGTTGGTATTGATTGTCCAAATGCAACTTGTATGGTAGTTGAGCTG

>MV307_*rplB*

AAAAACTTCATCTTTGCGTTATAAGACAACTTTATCTTTTGATGATTTAAGTAAGGGTAATAGTCCTTTGAAGTCTTTAACTAAGGGGAAGGTATCTAGGGCTGGAAGAGATTCTTCTGGAAGAATTAGTGTTAGAAGAAGGGGTGGAGGACATAAAAGACGGTATAGGGAAATTGACTTTGGAAGAAGAGATAAGTTTGATATACCTGCTCGAGTTGTGTCTATTGAATATGATCCAAATAGAAGTTCTAATATAGCTTTACTTGTTTATAAGGATGGAGATAAAAGATATATTATTGCTCCTAAAGGCATTAAAGTAGGTGATGTTTTACAAAGTGGTCCAAAGGCTCCAATAAGGGTTGGTAATTCTTTGCCGCTTGAAAACATTCCTGTTGGTAAGGCAGTACATAACATTGAACTTAATGTTGGAAGAGGTGGACAGCTTGTAAGAGGTGCTGGGGGATATGCCATGGTGCTTGCTTCTGAGGGAAATTATGTAACAGTTAAGCTTCCATCAGGTGAGGTTCGCATGGTTTTTAAGAAATGCATGGCAACTCTTGGAGAAGTTGGGAATGAAAATTATATTAATGTTTCTATTGGTAAGGCTGGTAAGAGTAGATGGCTTGGTAAAAGGCCTAAGGTAAGAGGTGTTGCCATGAATCCTGTCGATCACCCACATGGAGGTGGTGAGGGTAAGACTTCTGGTGGTCGTCATCT

>MV307_*uvrA*

TTTAATTGATGTTGGACTTTCTTATTTATATTTAGATAGGATGTCTGGTACCCTTTCAGGGGGGGAGGCTCAACGAATTAGGCTTGCTACTCAAATAGGTTCAGCTCTTGCTGGAGTTCTTTATATACTTGATGAACCTAGTATTGGATTGCATCAAAGAGATAATGAAAAGTTAATAAGTACTCTTATTAGTTTGAAAGAACTTGGCAATACAGTAATTGTTGTAGAGCATGATGAGCAAACTTTGCGTACTGCTGATTATATTGTCGATGTTGGGCCTGGAGCTGGTATTCATGGTGGAGAGATAGTTGCTCAGGGAACTTTAGCTGATATTTTGAATAATGAAAATAGTTTGACTGGAAAGTATTTAAGTGGTCAGCTTAAAATAGAAGTCCCAAAAACAAGGCGTAAGAAGGGCAAAGAAGAAATTTTGCTTTTAAATGCTAATAAAAACAATTTAAAGAATATTAATGTTTGCATTCCTTTGGGAATTTTTACTGTAATAACGGGAGTTTCTGGTAGTGGTAAAAGTACTCTTTTAAATGAAGTATTATATCCTGCTCTTGATAGTAGGTTGAAATTAAATACAAGTTATTTTGATGGGTTTGAGAATATCATTGGGTATGAACAAATTGACAAAATTATTCAGATAAATCAAAAGCCAATAGGCAAGACACCAAGATCAAATCCTGCAACTTATGTTGGGTTTTT

4. MLST for MV350 isolate

>MV350_*clpA*

AGTAAAATAAGAGAGCATATGATCATTGATGAAGAGCTTTTATCTGATTTGATATTAAACATAAAACTTTTAAGGATTAGATTTTTATTAAAAAATAATACTCTTGGTATTTTTATTTTAATGGGTTCCTCTGATATAAATAAGAGTAAACTTGCATCTATTTTATCAGAAGAGCTTAAGCTTCCTAAGTTTACTTTGGGAATGAGTGAGTATGGCGATTTTGATGGAATTAATAGATTAATAGGACCTGTATATGGAGCTGAGTTTTATGATGAACCTACTAAATTTTTTAAATTTTTAAGTAAAACTTCAAGCTCAATTATTTTCCTAGCAGATTTTGATAAATCTCCAAAGAGAGTCATAGATTTTTTTTTTGAAGGATTTAAGACTGGGAAACTTTTTGATAATTTTGGTAGAGATGTAAGTTTGTCAGATAGTATAATAATAATAGATATTAATGTGGATCACGGAGAACTTAATGGTATTGGATTTAAAAATGAGACAGTAAATAGCAGGAACCTACTAGAAAAGCGTTTTTCTAGTAGGTTCCTGGATTTAGTGGATCACATTTTTTTCTTTAGACCTGTAAATGAGAGTGATTTTAAAAGGGCTATTATCGAGGAGATGAATAATTTTGTTAAGATATTAAAGGCTGAAAAGGTTTA

>MV350_*clpX*

TGCTGTTTATAATCATTATAAAAGGATATTTAGGGGCAATAAGAACGAAAGTGGAGTTGAGCTTGAGAAATCTAATATATTGATTGTGGGTCCCACTGGAAGTGGTAAGACTTTGCTTGCAAAAAAGTTGGCAGCTGAGATGAATGTTCCATTTACAATTGCGGATGCTACAACGCTTACTGAAGCTGGATATGTAGGAGAAGATGTTGAGAACATTTTGCTTAAGCTCATTCATGCTGCGCATGGCGATGTAAGTTTCGCAGAACGAGGTATCATTTATATTGATGAAATAGATAAGATTGCAAAGAAAGGTGAAAATGTTTCAATTACAAGGGATGTTTCTGGAGAAGGTGTTCAACAATCTTTATTAAAGATAATTGAAGGAACTATTGCAAATGTTCCACCAAGGGGTGGGAGAAAACATCCTTATGAAGATACTATTGAAATTAATACACATAATATATTATTCATATGTGGTGGGGCTTTTGTAGGGCTTGAAAATATTATTAAGAAAAGAATTAATAAAAGCTCTATTGGATTTTCATCTGCTGGTAGGAAAGATGCCAAGGAAGATAATGTTTTGAAATATTTAGAGATGGAAGATTTGGTTAAATTTGGACTCATTCCAGAGTTTGTTGGTAGGCTTCCTGTGCATTCTTATCTTGAAAAGCTAGAAAAAGAAGATTTAATTAAAATATTAGTTGAACCTGAAAATTCTATTGTCAGACAATATTGTCATATGTTTAAGATGGATAATGTTGATCTGGTGTTTGAGAAGGATGCACTTGAAGTAATTGCAGAGGAAGCTATGATTAAAAATACAGGTGCAAGAGGTTTAAGATCTATTTTAGAAGAATTACTTAAAGATGT

>MV350_*nifS*

ATTGTGATTTCATCTGTAGCTAAATACTACGCAAACTATAATGCAAATGTTCACAGAAGCGGACATGAACTTACAATACAATCCAGTTTAAAAGTAGAAGAAACAAGAAAAATTGTAAAAAGATTTATTAACGCAGAGTCTTGCAAAAACATAATCTTTAATTCTGGAACAACAGATGGAACAAATACAGTAACAAATTCATTGCTATTCTCAAAACTTTTAAAAGAAAACGATGAAATCATAGTAACAATGATAGAACACAATAGCAATTTGCTTCCTTGGATAAATATTGCCAAATTCTTAAACTTAAAGATTAAACTTGCAAAATTTAACGAAATGGGCATTATTCAACCAGAACAAATAAAAAACTTAATTACAGATAAAACAAAAATTATTGCTATATCTGGCATAAGCAATATACTAGGCACCATACAAGATTTAGAAGCAATTGGAAAAATTGCTAAAGATAACAAAATCATTCTATCTGTAGACGCAGCTCAAATGGCACCACATATGAACATAGATGTAAATAAAATAAATTGTGATTTTTTAGTATTCTCAGGGCACAAAATGCTTGCTCCAACAGGAATTGGAGTCTTATATATATCAAATAAAATCATAGATAAACTTAATAGCCCCAAACTAGGAGGAAATGCTATAGAAGATATTTTTATAAAAAATGGAGAGCTTAATTTTAAACCCCTTGAATCTCCAAATAAATTTGAATCGGGAACACCAAATATTGCAGGCATTCATTG

>MV350_*pepX*

TAAATTTAGCAGGAGTTGTATACTTTAATAAAGATGGAATAATTAACTCAGAACTAATAACAATTGAAAATATTGGAATTATACCAAATGTAGCTATTCACCTAAACCGACAAGTCAATGAAGGATTTGCATATGATGCTCATGAACACATTGTAATCATTACAAGTCTTCAAAAGAGCATTAAAGAAAAAATTCTAGAAAAACTGCAAATATCCAAAAAAGATTTTCTATCATGCGACTTAATATTCACAGCAGCTGAACCTGCCAAAATCATAGGCAGCGAGGGTGAATTTTTAGCATCCAAAAACCTTGACAATAAATCGGGATGTCACGCCATCATGAATGCATTTATTCACACTAATAACAATAAAAACAAAGTAATTGTATTTTTCGACAATGAAGAGATTGGGTCTTTAACTTCCAGAGGAGCTGATTCAAAACTATTAACAGAAGTCCTAGAAAGAATTGATCATGTTTTAAATTTAGGAAAAGAAGAACACTTAATAAAACTAAATAACTCATTCAACATTTCAATGGATGGTGCACATGGGGCTCATCCAGGTTACATAGACAAACATGACCCAAGCTATCAAATCAGTCTAGGTAAAGGAGTAACTATTAAAAGCAATGCTAACTTCAAATATGCAACAACAGCAAATAGCTGCGCAAAACTAAAATTACTAGCTATGAAAAACAATATTAAAATTCAAGAAATAATAATGAAAGCAAATACCAATACTGGAAGCACAATTGGTCCAATTTCAAATTCACAAACAGGTATTGAAACCATAGATATTGGAACTCCAATGTGG

>MV350_*pyrG*

ATTGAGATTGGTGGTACTGTAGGGGATATGGAAAATATTTTGTTTATTGAGACAATGAGGCAGATAAGATATGAAATTGGGAGTGATAATATTGCTTTTATTCACTTGACTTATATTCCAAATCCTGTTGGGATAAATGAACAAAAGTCTAAACCTACACAACAGAGTGTAAAGACTTTAAATAAAGCTGGAATTTTTCCAGATTTAATTATTGCGAGAAATTCTCAACTTTTAACAAACCAGATAAGGCAAAAAATAGCAATGTTTTGTAATGTTGATGCTTCATCTATTATTGATAATGTTGATGTTTCTACTATTTATGAAATCCCTCTATCTTTTTATAAACAAGGCTTACATGAAATTTTGGGATCAAGATTAAAAATAAATGTTAAGCCCAAAGTGGATAGTCTTGATCGATTAGTTAATATAATAAAGAAAAATCTTGTATTTCCTAAGAAGGTAGTCAATATTGCTATTTGTGGTAAATATACTGAGCTTGGTGATTCTTATGCATCAATATTTGAGTCTTTAACGCATGTGTCTGCTAATTTAGATATGTTGGTTAAGACAACTGTAATTGATAGTACTAATTTTAATGAGGAGATGTTGAAGAATATAGATGGTATAGTAGTGCCTGGTGGTTTTGGTGGTAGAGGATATGAAGGTAAAATTCGTGCAATTAAATATGCTCGTGAAAATAATATTCCTTTTCTTGGTATTTGTCTTGGTATGCAACTTGCA

>MV350_*recG*

AGTTGAGGCTGGATATCAAGTTGCATTAATGGTGCCTACTGATCTTTTGGCTAAGCAACATTATAGTAATTTGTCAAGCATATTAAAAGATTTTAATGTTTCTATAGCTCTTTTAACTGGTAGTTTAAAGAAGAGAGATAGGGACGATGTTTTGAAAAAACTTCAGAGTGGGATTTATAGTTTAGTAATTGGGACTCATGCTATTTTTGATCAAGGAACAAAATTTAAAAAATTAGCTTATATTATTATTGATGAACAGCATAAATTTGGTGTTGAACAAAGAGAGGCCCTTAAAAATAAGGGAGAAGGAGTTGATGTTCTTTTAATGTCAGCGACTCCTATTCCTAGGAGCTTAGCTTTGACTCTTTTTGGTGATCTTCAGGTATCTTTAATTAAGAAAGGTCCTGCAGGTCGAATACCCGTTACTACTTATTTAGCGAAGCATGGAAATGAAAAAAAAGTGTATGAGTTTTTAAAAAATGAACTTGCAAAAGGGCATCAAGTTTATTTTGTTTATCCCCTAATATCATCTTCACAGAAGTTTGATTTAAAAGATGCTACTAGTATGTGTTTAACGCTTAAGAATATTTTTGTTGAGTATTCTGTTGCTATGATTCATTCTAAGCTTGAGTCTAATATCAAAGAAGAAATTATGCAGGATTTTTATTTGAGGAAAATAGATATTTTAGTTGCAACAAGTGTTATTGAAGTTGGTATTGATTGTCCAAATGCAACTTGTATGGTAGTTGAGCATG

>MV350_*rplB*

AAAAACTTCATCTTGCGTTATAAGACAACTTTATCTTTTGATGATTTAAGTAAGGGTAATAGTCCTTTGAAGTCTTTAACTAAGGGGAAGGTATCTAGGGCTGGAAGAGATTCTTCTGGAAGAATTAGTGTTAGAAGAAGGGGTGGAGGACATAAAAGACGGTATAGGGAAATTGACTTTGGAAGAAGAGATAAGTTTGATATACCTGCTCGAGTTGTGTCTATTGAATATGATCCAAATAGAAGTTCTAATATAGCTTTACTTGTTTATAAGGATGGAGATAAAAGATATATTATTGCTCCTAAAGGCATTAAAGTAGGTGATGTTTTACAAAGTGGTCCAAAGGCTCCAATAAGGGTTGGTAATTCTTTGCCGCTTGAAAACATTCCTGTTGGTAAGGCAGTACATAACATTGAACTTAATGTTGGAAGAGGTGGACAGCTTGTAAGAGGTGCTGGGGGATATGCCATGGTGCTTGCTTCTGAGGGAAATTATGTAACAGTTAAGCTTCCATCAGGTGAGGTTCGCATGGTTTTTAAGAAATGCATGGCAACTCTTGGAGAAGTTGGGAATGAAAATTATATTAATGTTTCTATTGGTAAGGCTGGTAAGAGTAGATGGCTTGGTAAAAGGCCTAAGGTAAGAGGTGTTGCCATGAATCCTGTCGATCACCCACATGGAGGTGGTGAGGGTAAGACTTCTGGTGGTCGTCATC

>MV350_*uvrA*

TTTTAATTGATGTTGGACTTTCTTATTTATATTTAGATAGGATGTCTGGTACCCTTTCAGGGGGGGAGGCTCAACGAATTAGGCTTGCTACTCAAATAGGTTCAGCTCTTGCTGGAGTTCTTTATATACTTGATGAACCTAGTATTGGATTGCATCAAAGAGATAATGAAAAGTTAATAAGTACTCTTATTAGTTTGAAAGAACTTGGCAATACAGTAATTGTTGTAGAGCATGATGAGCAAACTTTGCGTACTGCTGATTATATTGTCGATGTTGGGCCTGGAGCTGGTATTCATGGTGGAGAGATAGTTGCTCAGGGAACTTTAGCTGATATTTTGAATAATGAAAATAGTTTGACTGGAAAGTATTTAAGTGGTCAGCTTAAAATAGAAGTCCCAAAAACAAGGCGTAAGAAGGGCAAAGAAGAAATTTTGCTTTTAAATGCTAATAAAAACAATTTAAAGAATATTAATGTTTGCATTCCTTTGGGAATTTTTACTGTAATAACGGGAGTTTCTGGTAGTGGTAAAAGTACTCTTTTAAATGAAGTATTATATCCTGCTCTTGATAGTAGGTTGAAATTAAATACAAGTTATTTTGATGGGTTTGAGAATATCATTGGGTATGAACAAATTGACAAAATTATTCAGATAAATCAAAAGCCAATAGGCAAGACACCAAGATCAAATCCTGCAACTTATGTTGGGTTATT

5. MLST for MV565 isolate

>MV565_*clpA*

TCTGATTTGATATTAAACATAAAACTTTTAAGGATTAGATTTTTATTAAAAAATAATACTCTTGGTATTTTTATTTTAATGGGTTCCTCTGATATAAATAAGAGTAAACTTGCATCTATTTTATCAGAAGAGCTTAAGCTTCCTAAGTTTACTTTGGGAATGAGTGAGTATGGCGATTTTGATGGAATTAATAGATTAATAGGACCTGTATATGGAGCTGAGTTTTATGATGAACCTACTAAATTTTTTAAATTTTTAAGTAAAACTTCAAGCTCAATTATTTTCCTAGCAGATTTTGATAAATCTCCAAAGAGAGTCATAGATTTTTTTTTTGAAGGATTTAAGACTGGGAAACTTTTTGATAATTTTGGTAGAGATGTAAGTTTGTCAGATAGTATAATAATAATAGATATTAATGTGGATCACGGAGAACTTAATGGTATTGGATTTAAAAATGAGACAGTAAATAGCAGGAACCTACTAGAA

>MV565_*clpX*

GTGCTGTTTATAATCATTATAAAAGGATATTTAGGGGCAATAAGAACGAAAGTGGAGTTGAGCTTGAGAAATCTAATATATTGATTGTGGGTCCCACTGGAAGTGGTAAGACTTTGCTTGCAAAAAAGTTGGCAGCTGAGATGAATGTTCCATTTACAATTGCGGATGCTACAACGCTTACTGAAGCTGGATATGTAGGAGAAGATGTTGAGAACATTTTGCTTAAGCTCATTCATGCTGCGCATGGCGATGTAAGTTTCGCAGAACGAGGTATCATTTATATTGATGAAATAGATAAGATTGCAAAGAAAGGTGAAAATGTTTCAATTACAAGGGATGTTTCTGGAGAAGGTGTTCAACAATCTTTATTAAAGATAATTGAAGGAACTATTGCAAATGTTCCACCAAGGGGTGGGAGAAAACATCCTTATGAAGATACTATTGAAATTAATACACATAATATATTATTCATATGTGGTGGGGCTTTTGTAGGGCTTGAAAATATTATTAAGAAAAGAATTAATAAAAGCTCTATTGGATTTTCATCTGCTGGTAGGAAAGATGCCAAGGAAGATAATGTTTTGAAATATTTAGAGATGGAAGATTTGGTTAAATTTGGACTCATTCCAGAGTTTGTTGGTAGGCTTCCTGTGCATTCTTATCTTGAAAAGCTAGAAAAAGAAGATTTAATTAAAATATTAGTTGAACCTGAAAATTCTATTGTCAGACAATATTGTCATATGTTTAAGATGGATAATGTTGATCTGGTGTTTGAGAAGGATGCACTTGAAGTAATTGCAGAGGAAGCTATGATTAAAAATACAGGTGCAAGAGGT

>MV565_*pepX*

TAAATTTAGCAGGAGTTGTATACTTTAATAAAGATGGAATAATTAACTCAGAACTAATAACAATTGAAAATATTGGAATTATACCAAATGTAGCTATTCACCTAAACCGACAAGTCAATGAAGGATTTGCATATGATGCTCATGAACACATTGTAATCATTACAAGTCTTCAAAAGAGCATTAAAGAAAAAATTCTAGAAAAACTGCAAATATCCAAAAAAGATTTTCTATCATGCGACTTAATATTCACAGCAGCTGAACCTGCCAAAATCATAGGCAGCGAGGGTGAATTTTTAGCATCCAAAAACCTTGACAATAAATCGGGATGTCACGCCATCATGAATGCATTTATTCACACTAATAACAATAAAAACAAAGTAATTGTATTTTTCGACAATGAAGAGATTGGGTCTTTAACTTCCAGAGGAGCTGATTCAAAACTATTAACAGAAGTCCTAGAAAGAATTGATCATGTTTTAAATTTAGGAAAAGAAGAACACTTAATAAAACTAAATAACTCATTCAACATTTCAATGGATGGTGCACATGGGGCTCATCCAGGTTACATAGACAAACATGACCCAAGCTATCAAATCAGTCTAGGTAAAGGAGTAACTATTAAAAGCAATGCTAACTTCAAATATGCAACAACAGCAAATAGCTGCGCAAAACTAAAATTACTAGCTATGAAAAACAATATTAAAATTCAAGAAATAATAATGAAAGCAAATACCAATACTGGAAGCACAATTGGTCCAATTTCAAATTCACAAACAGGTATTGAAACCATAGATATTGGAACTCCAATGTGGGG

>MV565_*pyrG*

ATTGAGATTGGTGGTACTGTAGGGGATATGGAAAATATTTTGTTTATTGAGACAATGAGGCAGATAAGATATGAAATTGGGAGTGATAATATTGCTTTTATTCACTTGACTTATATTCCAAATCCTGTTGGGATAAATGAACAAAAGTCTAAACCTACACAACAGAGTGTAAAGACTTTAAATAAAGCTGGAATTTTTCCAGATTTAATTATTGCGAGAAATTCTCAACTTTTAACAAACCAGATAAGGCAAAAAATAGCAATGTTTTGTAATGTTGATGCTTCATCTATTATTGATAATGTTGATGTTTCTACTATTTATGAAATCCCTCTATCTTTTTATAAACAAGGCTTACATGAAATTTTGGGATCAAGATTAAAAATAAATGTTAAGCCCAAAGTGGATAGTCTTGATCGATTAGTTAATATAATAAAGAAAAATCTTGTATTTCCTAAGAAGGTAGTCAATATTGCTATTTGTGGTAAATATACTGAGCTTGGTGATTCTTATGCATCAATATTTGAGTCTTTAACGCATGTGTCTGCTAATTTAGATATGTTGGTTAAGACAACTGTAATTGATAGTACTAATTTTAATGAGGAGATGTTGAAGAATATAGATGGTATAGTAGTGCCTGGTGGTTTTGGTGGTAGAGGATATGAAGGTAAAATTCGTGCAATTAAATATGCTCGTGAAAATAATATTCCTTTTCT

>MV565_*recG*

TAGCATTCCTTTAGTTGAGGCTGGATATCAAGTTGCATTAATGGTGCCTACTGATCTTTTGGCTAAGCAACATTATAGTAATTTGTCAAGCATATTAAAAGATTTTAATGTTTCTATAGCTCTTTTAACTGGTAGTTTAAAGAAGAGAGATAGGGACGATGTTTTGAAAAAACTTCAGAGTGGGATTTATAGTTTAGTAATTGGGACTCATGCTATTTTTGATCAAGGAACAAAATTTAAAAAATTAGCTTATATTATTATTGATGAACAGCATAAATTTGGTGTTGAACAAAGAGAGGCCCTTAAAAATAAGGGAGAAGGAGTTGATGTTCTTTTAATGTCAGCGACTCCTATTCCTAGGAGCTTAGCTTTGACTCTTTTTGGTGATCTTCAGGTATCTTTAATTAAGAAAGGTCCTGCAGGTCGAATACCCGTTACTACTTATTTAGCGAAGCATGGAAATGAAAAAAAAGTGTATGAGTTTTTAAAAAATGAACTTGCAAAAGGGCATCAAGTTTATTTTGTTTATCCCCTAATATCATCTTCACAGAAGTTTGATTTAAAAGATGCTACTAGTATGTGTTTAACGCTTAAGAATATTTTTGTTGAGTATTCTGTTGCTATGATTCATTCTAAGCTTGAGTCTAATATCAAAGAAGAAATTATGCAGGATTTTTATTTGAGGAAAATAGATATTTTAGTTGCAACAAGTGTTATTGAAGTTGGTATTGATTGTCCAAATGCAACTTGTATGGTAGTTGAGCATGCTGAG

>MV565_*rplB*

GACTTTGGAAGAAGAGATAAGTTTGATATACCTGCTCGAGTTGTGTCTATTGAATATGATCCAAATAGAAGTTCTAATATAGCTTTACTTGTTTATAAGGATGGAGATAAAAGATATATTATTGCTCCTAAAGGCATTAAAGTAGGTGATGTTTTACAAAGTGGTCCAAAGGCTCCAATAAGGGTTGGTAATTCTTTGCCGCTTGAAAACATTCCTGTTGGTAAGGCAGTACATAACATTGAACTTAATGTTGGAAGAGGTGGACAGCTTGTAAGAGGTGCTGGGGGATATGCCATGGTGCTTGCTTCTGAGGGAAATTATGTAACAGTTAAGCTTCCATCAGGTGAGGTTCGCATGGTTTTTAAGAAATGCATGGCAACTCTTGGAGAAGTTGGGAATGAAAATTATATTAATGTTTCTATTGGTAAGGCTGGTAAGAGTAGATGGCTTGGTAAAAGGCCTAAGGTAAGAGGTGTTGCCATGAATCCTGTCGATCACCCACATGGAGGTGGTGAGGGTAAGACTTCTGGTGGTCGTCATC

>MV565_*uvrA*

TTTTAATTGATGTTGGACTTTCTTATTTATATTTAGATAGGATGTCTGGTACCCTTTCAGGGGGGGAGGCTCAACGAATTAGGCTTGCTACTCAAATAGGTTCAGCTCTTGCTGGAGTTCTTTATATACTTGATGAACCTAGTATTGGATTGCATCAAAGAGATAATGAAAAGTTAATAAGTACTCTTATTAGTTTGAAAGAACTTGGCAATACAGTAATTGTTGTAGAGCATGATGAGCAAACTTTGCGTACTGCTGATTATATTGTCGATGTTGGGCCTGGAGCTGGTATTCATGGTGGAGAGATAGTTGCTCAGGGAACTTTAGCTGATATTTTGAATAATGAAAATAGTTTGACTGGAAAGTATTTAAGTGGTCAGCTTAAAATAGAAGTCCCAAAAACAAGGCGTAAGAAGGGCAAAGAAGAAATTTTGCTTTTAAATGCTAATAAAAACAATTTAAAGAATATTAATGTTTGCATTCCTTTGGGAATTTTTACTGTAATAACGGGAGTTTCTGGTAGTGGTAAAAGTACTCTTTTAAATGAAGTATTATATCCTGCTCTTGATAGTAGGTTGAAATTAAATACAAGTTATTTTGATGGGTTTGAGAATATCATTGGGTATGAACAAATTGACAAAATTATTCAGATAAATCAAAAGCCAATAGGCAAGACACCAAGATCAAATCCTGCAACTTATGTTGGGTTTTTT

6. MLST for MV589 isolate

>MV589_*clpA*

TAATCTGGAGAGTAAAATAAGAGAGCATATGATCATTGATGAAGAGCTTTTATCTGATTTGATATTAAACATAAAACTTTTAAGGATTAGATTTTTATTAAAAAATAATACTCTTGGTATTTTTATTTTAATGGGTTCCTCTGATATAAATAAGAGTAAACTTGCATCTATTTTATCAGAAGAGCTTAAGCTTCCTAAGTTTACTTTGGGAATGAGTGAGTATGGCGATTTTGATGGAATTAATAGATTAATAGGACCTGTATATGGAGCTGAGTTTTATGATGAACCTACTAAATTTTTTAAATTTTTAAGTAAAACTTCAAGCTCAATTATTTTCCTAGCAGATTTTGATAAATCTCCAAAGAGAGTCATAGATTTTTTTTTTGAAGGATTTAAGACTGGGAAACTTTTTGATAATTTTGGTAGAGATGTAAGTTTGTCAGATAGTATAATAATAATAGATATTAATGTGGATCACGGAGAACTTAATGGTATTGGATTTAAAAATGAGACAGTAAAT

>MV589_*clpX*

GCTGTTTATAATCATTATAAAAGGATATTTAGGGGCAATAAGAACGAAAGTGGAGTTGAGCTTGAGAAATCTAATATATTGATTGTGGGTCCCACTGGAAGTGGTAAGACTTTGCTTGCAAAAAAGTTGGCAGCTGAGATGAATGTTCCATTTACAATTGCGGATGCTACAACGCTTACTGAAGCTGGATATGTAGGAGAAGATGTTGAGAACATTTTGCTTAAGCTCATTCATGCTGCGCATGGCGATGTAAGTTTCGCAGAACGAGGTATCATTTATATTGATGAAATAGATAAGATTGCAAAGAAAGGTGAAAATGTTTCAATTACAAGGGATGTTTCTGGAGAAGGTGTTCAACAATCTTTATTAAAGATAATTGAAGGAACTATTGCAAATGTTCCACCAAGGGGTGGGAGAAAACATCCTTATGAAGATACTATTGAAATTAATACACATAATATATTATTCATATGTGGTGGGGCTTTTGTAGGGCTTGAAAATATTATTAAGAAAAGAATTAATAAAAGCTCTATTGGATTTTCATCTGCTGGTAGGAAAGATGCCAAGGAAGATAATGTTTTGAAATATTTAGAGATGGAAGATTTGGTTAAATTTGGACTCATTCCAGAGTTTGTTGGTAGGCTTCCTGTGCATTCTTATCTTGAAAAGCTAGAAAAAGAAGATTTAATTAAAATATTAGTTGAACCTGAAAATTCTATTGTCAGACAATATTGTCATATGTTTAAGATGGATAATGTTGATCTGGTGTTTGAGAAGGATGCACTTGAAGTAATTGCAGAGGAAGCTATGATTAAAAATACAGGTGCAAGAGGTTTAAGATCTATTTTAGAAGAATTACTTAAAGATGTTATGT

>MV589_*nifS*

CAATACAATCCAGTTTAAAAGTAGAAGAAACAAGAAAAATTGTAAAAAGATTTATTAACGCAGAGTCTTGCAAAAACATAATCTTTAATTCTGGAACAACAGATGGAACAAATACAGTAACAAATTCATTGCTATTCTCAAAACTTTTAAAAGAAAACGATGAAATCATAGTAACAATGATAGAACACAATAGCAATTTGCTTCCTTGGATAAATATTGCCAAATTCTTAAACTTAAAGATTAAACTTGCAAAATTTAACGAAATGGGCATTATTCAACCAGAACAAATAAAAAACTTAATTACAGATAAAACAAAAATTATTGCTATATCTGGCATAAGCAATATACTAGGCACCATACAAGATTTAGAAGCAATTGGAAAAATTGCTAAAGATAACAAAATCATTCTATCTGTAGACGCAGCTCAAATGGCACCACATATGAACATAGATGTAAATAAAATAAATTGTGATTTTTTAGTATTCTCAGGGCACAAAATGCTTGCTCCAACA

>MV589_*pepX*

TAAATTTAGCAGGAGTTGTATACTTTAATAAAGATGGAATAATTAACTCAGAACTAATAACAATTGAAAATATTGGAATTATACCAAATGTAGCTATTCACCTAAACCGACAAGTCAATGAAGGATTTGCATATGATGCTCATGAACACATTGTAATCATTACAAGTCTTCAAAAGAGCATTAAAGAAAAAATTCTAGAAAAACTGCAAATATCCAAAAAAGATTTTCTATCATGCGACTTAATATTCACAGCAGCTGAACCTGCCAAAATCATAGGCAGCGAGGGTGAATTTTTAGCATCCAAAAACCTTGACAATAAATCGGGATGTCACGCCATCATGAATGCATTTATTCACACTAATAACAATAAAAACAAAGTAATTGTATTTTTCGACAATGAAGAGATTGGGTCTTTAACTTCCAGAGGAGCTGATTCAAAACTATTAACAGAAGTCCTAGAAAGAATTGATCATGTTTTAAATTTAGGAAAAGAAGAACACTTAATAAAACTAAATAACTCATTCAACATTTCAATGGATGGTGCACATGGGGCTCATCCAGGTTACATAGACAAACATGACCCAAGCTATCAAATCAGTCTAGGTAAAGGAGTAACTATTAAAAGCAATGCTAACTTCAAATATGCAACAACAGCAAATAGCTGCGCAAAACTAAAATTACTAGCTATGAAAAACAATATTAAAATTCAAGAAATAATAATGAAAGCAAATACCAATACTGGAAGCACAATTGGTCCAATTTCAAATTCACAAACAGGTATTGAAACCATAGATATTGGAACTCCAATGTGGG

>MV589_*pyrG*

ATTGAGATTGGTGGTACTGTAGGGGATATGGAAAATATTTTGTTTATTGAGACAATGAGGCAGATAAGATATGAAATTGGGAGTGATAATATTGCTTTTATTCACTTGACTTATATTCCAAATCCTGTTGGGATAAATGAACAAAAGTCTAAACCTACACAACAGAGTGTAAAGACTTTAAATAAAGCTGGAATTTTTCCAGATTTAATTATTGCGAGAAATTCTCAACTTTTAACAAACCAGATAAGGCAAAAAATAGCAATGTTTTGTAATGTTGATGCTTCATCTATTATTGATAATGTTGATGTTTCTACTATTTATGAAATCCCTCTATCTTTTTATAAACAAGGCTTACATGAAATTTTGGGATCAAGATTAAAAATAAATGTTAAGCCCAAAGTGGATAGTCTTGATCGATTAGTTAATATAATAAAGAAAAATCTTGTATTTCCTAAGAAGGTAGTCAATATTGCTATTTGTGGTAAATATACTGAGCTTGGTGATTCTTATGCATCAATATTTGAGTCTTTAACGCATGTGTCTGCTAATTTAGATATGTTGGTTAAGACAACTGTAATTGATAGTACTAATTTTAATGAGGAGATGTTGAAGAATATAGATGGTATAGTAGTGCCTGGTGGTTTTGGTGGTAGAGGATATGAAGGTAAAATTCGTGCAATTAAATATGCTCGTGAAAATAATATTCCTTTTCTTGGTATTTGTCTTGGTATGCAACTTGCAGTAATTGAATTC

>MV589_*recG*

AGTTGAGGCTGGATATCAAGTTGCATTAATGGTGCCTACTGATCTTTTGGCTAAGCAACATTATAGTAATTTGTCAAGCATATTAAAAGATTTTAATGTTTCTATAGCTCTTTTAACTGGTAGTTTAAAGAAGAGAGATAGGGACGATGTTTTGAAAAAACTTCAGAGTGGGATTTATAGTTTAGTAATTGGGACTCATGCTATTTTTGATCAAGGAACAAAATTTAAAAAATTAGCTTATATTATTATTGATGAACAGCATAAATTTGGTGTTGAACAAAGAGAGGCCCTTAAAAATAAGGGAGAAGGAGTTGATGTTCTTTTAATGTCAGCGACTCCTATTCCTAGGAGCTTAGCTTTGACTCTTTTTGGTGATCTTCAGGTATCTTTAATTAAGAAAGGTCCTGCAGGTCGAATACCCGTTACTACTTATTTAGCGAAGCATGGAAATGAAAAAAAAGTGTATGAGTTTTTAAAAAATGAACTTGCAAAAGGGCATCAAGTTTATTTTGTTTATCCCCTAATATCATCTTCACAGAAGTTTGATTTAAAAGATGCTACTAGTATGTGTTTAACGCTTAAGAATATTTTTGTTGAGTATTCTGTTGCTATGATTCATTCTAAGCTTGAGTCTAATATCAAAGAAGAAATTATGCAGGATTTTTATTTGAGGAAAATAGATATTTTAGTTGCAACAAGTGTTA

>MV589_*rplB*

GGCCAAAAACTTCATCTTTGCGTTATAAGACAACTTTATCTTTTGATGATTTAAGTAAGGGTAATAGTCCTTTGAAGTCTTTAACTAAGGGGAAGGTATCTAGGGCTGGAAGAGATTCTTCTGGAAGAATTAGTGTTAGAAGAAGGGGTGGAGGACATAAAAGACGGTATAGGGAAATTGACTTTGGAAGAAGAGATAAGTTTGATATACCTGCTCGAGTTGTGTCTATTGAATATGATCCAAATAGAAGTTCTAATATAGCTTTACTTGTTTATAAGGATGGAGATAAAAGATATATTATTGCTCCTAAAGGCATTAAAGTAGGTGATGTTTTACAAAGTGGTCCAAAGGCTCCAATAAGGGTTGGTAATTCTTTGCCGCTTGAAAACATTCCTGTTGGTAAGGCAGTACATAACATTGAACTTAATGTTGGAAGAGGTGGACAGCTTGTAAGAGGTGCTGGGGGATATGCCATGGTGCTTGCTTCTGAGGGAAATTATGTAACAGTTAAGCTTCCATCAGGTGAGGTTCGCATGGTTTTTAAGAAATGCATGGCAACTCTTGGAGAAGTTGGGAATGAAAATTATATTAATGTTTCTATTGGTAAGGCTGGTAAGAGTAGATGGCTTGGTAAAAGGCCTAAGGTAAGAGGTGTTGCCATGAATCCTGTCGATCACCCACATGGAGGTGGTGAGGGTAAGACTTCTGGTGGTCGTCATCT

>MV589_*uvrA*

TTTTAATTGATGTTGGACTTTCTTATTTATATTTAGATAGGATGTCTGGTACCCTTTCAGGGGGGGAGGCTCAACGAATTAGGCTTGCTACTCAAATAGGTTCAGCTCTTGCTGGAGTTCTTTATATACTTGATGAACCTAGTATTGGATTGCATCAAAGAGATAATGAAAAGTTAATAAGTACTCTTATTAGTTTGAAAGAACTTGGCAATACAGTAATTGTTGTAGAGCATGATGAGCAAACTTTGCGTACTGCTGATTATATTGTCGATGTTGGGCCTGGAGCTGGTATTCATGGTGGAGAGATAGTTGCTCAGGGAACTTTAGCTGATATTTTGAATAATGAAAATAGTTTGACTGGAAAGTATTTAAGTGGTCAGCTTAAAATAGAAGTCCCAAAAACAAGGCGTAAGAAGGGCAAAGAAGAAATTTTGCTTTTAAATGCTAATAAAAACAATTTAAAGAATATTAATGTTTGCATTCCTTTGGGAATTTTTACTGTAATAACGGGAGTTTCTGGTAGTGGTAAAAGTACTCTTTTAAATGAAGTATTATATCCTGCTCTTGATAGTAGGTTGAAATTAAATACAAGTTATTTTGATGGGTTTGAGAATATCATTGGGTATGAACAAATTGACAAAATTATTCAGATAAATCAAAAGCCAATAGGCAAGACACCAAGATCAAATCCTGCAACTTATGTTGGGTTTTT

7. MLST for MV591 isolate

>MV591_*clpA*

TGAATTACTTATTAATCTGGAGAGTAAAATAAGAGAGCATATGATCATTGATGAAGAGCTTTTATCTGATTTGATATTAAACATAAAACTTTTAAGGATTAGATTTTTATTAAAAAATAATACTCTTGGTATTTTTATTTTAATGGGTTCCTCTGATATAAATAAGAGTAAACTTGCATCTATTTTATCAGAAGAGCTTAAGCTTCCTAAGTTTACTTTGGGAATGAGTGAGTATGGCGATTTTGATGGAATTAATAGATTAATAGGACCAGTATAAGGAGCTGAGTTTTATGATGAACCTACTAAATTTTTTAAATTTTTAAGTAAAACTTCAAGCTCAATTATTTTCCTAGCAGATTTTGATAAATCTCCAAAGAGAGTCATAGATTTTTTTTTTGAAGGATTTAAGACTGGGAAACTTTTTGATAATTTTGGTAGAGATGTAAGTTTGTCAGATAGTATAATAATAATAGATATTAATGTGGATCACGGAGAA

>MV591_*clpX*

GTGCTGTTTATAATCATTATAAAAGGATATTTAGGGGCAATAAGAACGAAAGTGGAGTTGAGCTTGAGAAATCTAATATATTGATTGTGGGTCCCACTGGAAGTGGTAAGACTTTGCTTGCAAAAAAGTTGGCAGCTGAGATGAATGTTCCATTTACAATTGCGGATGCTACAACGCTTACTGAAGCTGGATATGTAGGAGAAGATGTTGAGAACATTTTGCTTAAGCTCATTCATGCTGCGCATGGCGATGTAAGTTTCGCAGAACGAGGTATCATTTATATTGATGAAATAGATAAGATTGCAAAGAAAGGTGAAAATGTTTCAATTACAAGGGATGTTTCTGGAGAAGGTGTTCAACAATCTTTATTAAAGATAATTGAAGGAACTATTGCAAATGTTCCACCAAGGGGTGGGAGAAAACATCCTTATGAAGATACTATTGAAATTAATACACATAATATATTATTCATATGTGGTGGGGCTTTTGTAGGGCTTGAAAATATTATTAAGAAAAGAATTAATAAAAGCTCTATTGGATTTTCATCTGCTGGTAGGAAAGATGCCAAGGAAGATAATGTTTTGAAATATTTAGAGATGGAAGATTTGGTTAAATTTGGACTCATTCCAGAGTTTGTTGGTAGGCTTCCTGTGCATTCTTATCTTGAAAAGCTAGAAAAAGAAGATTTAATTAAAATATTAGTTGAACCTGAAAATTCTATTGTCAGACAATATTGTCATATGTTTAAGATGGATAATGTTGATCTGGTGTTTGAGAAGGATGCACTTGAAGTAATTGCAGAGGAAGCTATGATTAAAAATACAGGTGCAAGAGGTTTAAGATCTATTTT

>MV591_*pepX*

TAAATTTAGCAGGAGTTGTATACTTTAATAAAGATGGAATAATTAACTCAGAACTAATAACAATTGAAAATATTGGAATTATACCAAATGTAGCTATTCACCTAAACCGACAAGTCAATGAAGGATTTGCATATGATGCTCATGAACACATTGTAATCATTACAAGTCTTCAAAAGAGCATTAAAGAAAAAATTCTAGAAAAACTGCAAATATCCAAAAAAGATTTTCTATCATGCGACTTAATATTCACAGCAGCTGAACCTGCCAAAATCATAGGCAGCGAGGGTGAATTTTTAGCATCCAAAAACCTTGACAATAAATCGGGATGTCACGCCATCATGAATGCATTTATTCACACTAATAACAATAAAAACAAAGTAATTGTATTTTTCGACAATGAAGAGATTGGGTCTTTAACTTCCAGAGGAGCTGATTCAAAACTATTAACAGAAGTCCTAGAAAGAATTGATCATGTTTTAAATTTAGGAAAAGAAGAACACTTAATAAAACTAAATAACTCATTCAACATTTCAATGGATGGTGCACATGGGGCTCATCCAGGTTACATAGACAAACATGACCCAAGCTATCAAATCAGTCTAGGTAAAGGAGTAACTATTAAAAGCAATGCTAACTTCAAATATGCAACAACAGCAAATAGCTGCGCAAAACTAAAATTACTAGCTATGAAAAACAATATTAAAATTCAAGAAATAATAATGAAAGCAAATACCAATACTGGAAGCACAATTGGTCCAATTTCAAATTCACAAACAGGTATTGAAACCATAGATATTGGAACTCCAATGTGGGGAATGCAA

>MV591_*pyrG*

ATTGAGATTGGTGGTACTGTAGGGGATATGGAAAATATTTTGTTTATTGAGACAATGAGGCAGATAAGATATGAAATTGGGAGTGATAATATTGCTTTTATTCACTTGACTTATATTCCAAATCCTGTTGGGATAAATGAACAAAAGTCTAAACCTACACAACAGAGTGTAAAGACTTTAAATAAAGCTGGAATTTTTCCAGATTTAATTATTGCGAGAAATTCTCAACTTTTAACAAACCAGATAAGGCAAAAAATAGCAATGTTTTGTAATGTTGATGCTTCATCTATTATTGATAATGTTGATGTTTCTACTATTTATGAAATCCCTCTATCTTTTTATAAACAAGGCTTACATGAAATTTTGGGATCAAGATTAAAAATAAATGTTAAGCCCAAAGTGGATAGTCTTGATCGATTAGTTAATATAATAAAGAAAAATCTTGTATTTCCTAAGAAGGTAGTCAATATTGCTATTTGTGGTAAATATACTGAGCTTGGTGATTCTTATGCATCAATATTTGAGTCTTTAACGCATGTGTCTGCTAATTTAGATATGTTGGTTAAGACAACTGTAATTGATAGTACTAATTTTAATGAGGAGATGTTGAAGAATATAGATGGTATAGTAGTGCCTGGTGGTTTTGGTGGTAGAGGATATGAAGGTAAAATTCGTGCAATTAAATATGCTCGTGAAAATAATATTCCTTTTCT

>MV591_*recG*

TTCCTTTAGTTGAGGCTGGATATCAAGTTGCATTAATGGTGCCTACTGATCTTTTGGCTAAGCAACATTATAGTAATTTGTCAAGCATATTAAAAGATTTTAATGTTTCTATAGCTCTTTTAACTGGTAGTTTAAAGAAGAGAGATAGGGACGATGTTTTGAAAAAACTTCAGAGTGGGATTTATAGTTTAGTAATTGGGACTCATGCTATTTTTGATCAAGGAACAAAATTTAAAAAATTAGCTTATATTATTATTGATGAACAGCATAAATTTGGTGTTGAACAAAGAGAGGCCCTTAAAAATAAGGGAGAAGGAGTTGATGTTCTTTTAATGTCAGCGACTCCTATTCCTAGGAGCTTAGCTTTGACTCTTTTTGGTGATCTTCAGGTATCTTTAATTAAGAAAGGTCCTGCAGGTCGAATACCCGTTACTACTTATTTAGCGAAGCATGGAAATGAAAAAAAAGTGTATGAGTTTTTAAAAAATGAACTTGCAAAAGGGCATCAAGTTTATTTTGTTTATCCCCTAATATCATCTTCACAGAAGTTTGATTTAAAAGATGCTACTAGTATGTGTTTAACGCTTAAGAATATTTTTGTTGAGTATTCTGTTGCTATGATTCATTCTAAGCTTGAGTCTAATATCAAAGAAGAAATTATGCAGGATTTTTATTTGAGGAAAATAGATATTTTAGTTGCAACAAGTGTTA

>MV591_*rplB*

AAAAACTTCATCTTGCGTTATAAGACAACTTTATCTTTTGATGATTTAAGTAAGGGTAATAGTCCTTTGAAGTCTTTAACTAAGGGGAAGGTATCTAGGGCTGGAAGAGATTCTTCTGGAAGAATTAGTGTTAGAAGAAGGGGTGGAGGACATAAAAGACGGTATAGGGAAATTGACTTTGGAAGAAGAGATAAGTTTGATATACCTGCTCGAGTTGTGTCTATTGAATATGATCCAAATAGAAGTTCTAATATAGCTTTACTTGTTTATAAGGATGGAGATAAAAGATATATTATTGCTCCTAAAGGCATTAAAGTAGGTGATGTTTTACAAAGTGGTCCAAAGGCTCCAATAAGGGTTGGTAATTCTTTGCCGCTTGAAAACATTCCTGTTGGTAAGGCAGTACATAACATTGAACTTAATGTTGGAAGAGGTGGACAGCTTGTAAGAGGTGCTGGGGGATATGCCATGGTGCTTGCTTCTGAGGGAAATTATGTAACAGTTAAGCTTCCATCAGGTGAGGTTCGCATGGTTTTTAAGAAATGCATGGCA

>MV591_*uvrA*

TTTTAATTGATGTTGGACTTTCTTATTTATATTTAGATAGGATGTCTGGTACCCTTTCAGGGGGGGAGGCTCAACGAATTAGGCTTGCTACTCAAATAGGTTCAGCTCTTGCTGGAGTTCTTTATATACTTGATGAACCTAGTATTGGATTGCATCAAAGAGATAATGAAAAGTTAATAAGTACTCTTATTAGTTTGAAAGAACTTGGCAATACAGTAATTGTTGTAGAGCATGATGAGCAAACTTTGCGTACTGCTGATTATATTGTCGATGTTGGGCCTGGAGCTGGTATTCATGGTGGAGAGATAGTTGCTCAGGGAACTTTAGCTGATATTTTGAATAATGAAAATAGTTTGACTGGAAAGTATTTAAGTGGTCAGCTTAAAATAGAAGTCCCAAAAACAAGGCGTAAGAAGGGCAAAGAAGAAATTTTGCTTTTAAATGCTAATAAAAACAATTTAAAGAATATTAATGTTTGCATTCCTTTGGGAATTTTTACTGTAATAACGGGAGTTTCTGGTAGTGGTAAAAGTACTCTTTTAAATGAAGTATTATATCCTGCTCTTGATAGTAGGTTGAAATTAAATACAAGTTATTTTGATGGGTTTGAGAATATCATTGGGTATGAACAAATTGACAAAATTATTCAGATAAATCAAAAGCCAATAGGCAAGACACCAAGATCAAATCCTGCAACTTATGTTGGGTTTTT
